# Population pharmacokinetic/pharmacodynamic modeling to optimize aztreonam-avibactam dose regimens for adult patients

**DOI:** 10.1128/aac.01950-24

**Published:** 2025-06-18

**Authors:** Rujia Xie, Halley Rogers, Joseph W. Chow, Elena Soto, Susan R. Raber

**Affiliations:** 1 Pfizer Ltd 68725 , Singapore, Singapore; 2 Pfizer Inc 2253 , New York, New York, USA; 3 Pfizer Inc 462954 , Collegeville, Pennsylvania, USA; 4 Pfizer Inc, Sandwich, Kent, United Kingdom; 5 Pfizer Inc 17475 , La Jolla, California, USA; Providence Portland Medical Center, Portland, Oregon, USA

**Keywords:** aztreonam-avibactam, population pharmacokinetics, pharmacodynamics

## Abstract

Aztreonam-avibactam, a fixed-ratio (3:1) β-lactam/β-lactamase inhibitor combination, was approved in Europe in 2024 for adults with complicated intra-abdominal infection (cIAI), complicated urinary tract infection, hospital-acquired/ventilator-associated pneumonia, and other infections due to aerobic gram-negative organisms with limited treatment options. Pharmacokinetic data from two phase 3 trials were added to previous data to develop a simultaneous aztreonam and avibactam population pharmacokinetic model. The final model was used to simulate exposures by infection type and renal function and estimate the joint probability of pharmacodynamic target attainment (PTA) for phase 3 patients (aztreonam 60% *f*T >MIC_ATM-AVI_ of 8 mg/L and avibactam 50% *f*T >C_T_ of 2.5 mg/L). Additional simulations evaluated simplified loading doses, regimens for end-stage renal disease, and ceftazidime-avibactam + aztreonam regimens. The final model included 4,914 aztreonam plasma samples (431 subjects) and 18,222 avibactam plasma samples (2,635 subjects). A two-compartment model with zero-order infusion and first-order elimination best fit the data. Time-varying creatinine clearance was a key covariate on clearance for both drugs, whereas infection type was a key covariate on clearance and volume, with the lowest exposures observed in patients with cIAI. Final aztreonam-avibactam dose regimens achieved joint PTA 89% to >99% at steady state across renal function groups. Ceftazidime-avibactam + aztreonam dose regimens proposed by the Infectious Diseases Society of America (IDSA) achieved joint PTA <85% due to insufficient avibactam exposures. Approved aztreonam-avibactam dose regimens (single loading dose and regular maintenance doses, all 3 h intravenous infusions) are optimized for joint PTA across renal function groups and infection types.

**CLINICAL TRIALS:**

This study is registered with ClinicalTrials.gov as NCT03329092 and NCT03580044.

## INTRODUCTION

Infections caused by carbapenem-resistant gram-negative pathogens, such as metallo-β-lactamase (MBL)-producing *Enterobacterales*, are an urgent global health problem for which treatment options are severely limited (1–3). Aztreonam-avibactam is a fixed-ratio (3:1) β-lactam (monobactam)/β-lactamase inhibitor combination developed for the treatment of serious infections caused by gram-negative bacteria, including serine carbapenemase- and MBL-producing *Enterobacterales* (4–6).

Aztreonam-avibactam was approved in April 2024 in Europe for the treatment of adults with complicated intra-abdominal infection (cIAI), complicated urinary tract infection (cUTI), and hospital-acquired pneumonia (HAP), including ventilator-associated pneumonia (VAP), and for the treatment of other infections due to aerobic gram-negative organisms in adult patients with limited treatment options (7). The approval was supported by an adult clinical program including one phase 2a and two phase 3 trials (4–6) and includes recommended dosing regimens (single loading doses followed by regular maintenance doses, all given as 3 h intravenous infusions).

Optimizing dosing schedules is paramount to ensuring the effectiveness of antimicrobial therapies, particularly for seriously ill patient populations, in which substantial co-morbidities and renal dysfunction are common, and pharmacokinetic (PK) variability is typically wider than in healthy subjects (8–10). In general, dose selection for antibiotic/inhibitor combinations is based on achievement of a joint pharmacokinetic/pharmacodynamic (PK/PD) target in simulated patient populations, together with consideration of clinical efficacy and safety data and microbiological activity against relevant target pathogen(s) (11). For the aztreonam-avibactam combination, the primary PK/PD target derived against MBL-producing gram-negative pathogens from *in vitro* studies and animal models is simultaneous achievement of free aztreonam concentration above a nominal aztreonam-avibactam MIC value of 8 mg/L for 60% of the dosing interval (60% *f*T >MIC_ATM-AVI_ of 8 mg/L) and free avibactam concentration above a threshold of 2.5 mg/L for 50% of the dosing interval (50% *f*T >C_T_ of 2.5 mg/L) (11–13).

The aztreonam-avibactam phase 3 dose regimen comprised intravenous infusions of a 30 min loading dose (LD), immediately followed by a 3 h extended loading dose (ELD), with 3 h maintenance doses (MD) administered every 6 h (q6h) with adjustments for patients with moderate or severe renal impairment (12). The phase 3 trials did not enroll patients with end-stage renal disease (ESRD) requiring dialysis or patients receiving continuous renal replacement therapy. Initial analyses to support phase 3 doses modeled aztreonam and avibactam separately (12). A simultaneous aztreonam and avibactam population PK model was subsequently developed to efficiently include estimated correlations of PK variability of the two drugs for parametric simulations and was used to support dose selection for pediatric evaluation of aztreonam-avibactam (14).

Following completion of the adult phase 3 trials (5, 6) and two further phase 1 studies (15, 16), an updated simultaneous aztreonam and avibactam population PK model was developed to evaluate exposures in phase 3 patients, confirm dose regimen recommendations across renal function groups including augmented renal clearance (ARC) and ESRD, and propose a simplified loading dose (SLD) for use in clinical practice. In addition, given that the Infectious Diseases Society of America (IDSA) has issued dosing recommendations for combination treatment with ceftazidime-avibactam + aztreonam (17), the final model was used to simulate comparative exposures and PK/PD target attainment for two proposed ceftazidime-avibactam + aztreonam regimens.

## MATERIALS AND METHODS

### Study data

This analysis included subject-level PK data from the previously reported (non-simultaneous) population PK analysis (12) and data sets from five additional adult clinical studies (two phase 1, one phase 2a, and two phase 3) of aztreonam-avibactam (4–6, 15, 16). Design details, including PK sampling schedules for these additional studies, are shown in Table 1. Bioanalytical methods for the studies included in the previous (non-simultaneous) population PK analysis have been reported (lower limits of quantification [LLOQs] were 0.04–0.1 mg/L for aztreonam and 0.01 mg/L for avibactam) (12). For four of the five additional studies, plasma samples for aztreonam and avibactam concentrations were analyzed by Labcorp Bioanalytical Services (Shanghai, China and Indianapolis, USA). Bioanalysis of plasma samples from the other study was performed by Covance Analytical Laboratories Ltd (Harrogate, UK). LLOQs for all five studies were 0.1 mg/L for aztreonam and 0.01 mg/L for avibactam.

**TABLE 1:** Overview of clinical studies added in the aztreonam-avibactam simultaneous population PK model^*a*^^,*b*,*c*,*d*,*e*,*f*,*g*,*h*,*i*,*j*,*k*,*l*,*m*,*n*,*o*,*p*^

Study	Design	Population	Treatments and duration	PK sampling schedule
C3601006 NCT04486625 (15)	Phase 1, open-label, parallel-group	Subjects with severe renal impairment (not on dialysis) (cohort 1) and healthy subjects with normal renal function (cohort 2)	Aztreonam-avibactam 4 days	Day 1: pre-dose, 0.5 (within 5 min before the end of LD) and 3.5 (within 15 min before end of ELD) h after start of the LD, pre-dose (within 10 min before start of each MD) Day 2: pre-dose (within 10 min before start of each MD) Day 3: pre-dose (within 10 min before start of each MD), and intensive PK samples were collected after final MD at pre-dose, 2, 3, 3.25, 3.5, 3.75, 4, 5, 6, 8, 12, 16, and 24 h after the start of the IV infusion
C3601007 NCT04973826 (16)	Phase 1, open-label	Healthy Chinese subjects	Aztreonam-avibactam 5 days (single and multiple doses)	Day 1: pre-dose, 2, 3 (15 min before IV infusion stop), 3.25, 3.5, 4, 5, 6, 8, 12, and 16 h after a single dose Day 2: pre-dose, 0.5, and 3.5 h after the start of the LD, and before the start of 2nd MD Day 3: before the start of 4th MD Day 4: pre-dose, 2, 3 (15 min before IV infusion stop), 3.25, 3.5, 4, 5, 6, 8, 12, 16, and 24 h
C3601001 NCT02655419 (REJUVENATE) (4)	Phase 2a, prospective, open-label	Hospitalized adults with cIAI	Aztreonam-avibactam *+*metronidazole 5–14 days	Day 1 (four samples/patient): pre-dose, within 5 min before the end of the LD infusion, 3.25–3.5 h and 5–6 h after start of the LD infusion Day 4 (intensive sampling for the first 25 patients; 11 samples/patient): Trough (within 10 min prior to MD infusion start), 0.5, 1, 2, 3 (within 15 min before MD infusion stop), 3.25, 3.5, 3.75, 4, 5, and 6 h after start of MD infusion (just before start of next infusion) Day 4 (sparse sampling for remaining patients; three samples/patient): Within 1 h, 2.75–3 h (within 15 min before MD infusion stop), and 5–6 h (before start of next MD) after start of MD infusion
C3601002 NCT03329092 (REVISIT) (5)	Phase 3, prospective, randomized, open-label, parallel group, comparative	Hospitalized adults with cIAI or NP (HAP/VAP)	Aztreonam-avibactam *±*metronidazole Meropenem *±* colistin 5–14 days for cIAI 7–14 days for HAP/VAP	All subjects randomized to aztreonam-avibactam had a a total of six blood samples collected Day 1: 25–30 min, 3.25–3.5 h, and 5.5–6.5 h (for q6h) or 7.5–8.5 h (for q8h) after the start of the LD Day 4: 2.75–3 h, 3.5–4.5 h, and 5–6 h (for q6h) or 7–8 h (for q8h) after the start of the IV infusion
C3601009 NCT03580044 (ASSEMBLE) (6)	Phase 3, prospective, randomized, open-label, parallel group, comparative	Hospitalized adults with cIAI, NP (HAP/VAP), cUTI, or BSI (MBL-producing Gram-negative pathogens)	Aztreonam-avibactam *±* metronidazole BAT 5–14 days for cIAI/cUTI/BSI 7–14 days for HAP/VAP	All subjects randomized to aztreonam-avibactam had total of six blood samples collected Day 1: 25–30 min, 3.25–3.5 h, and 5.5–6.5 h (for q6h) or 7.5–8.5 h (for q8h) after the start of the LD Day 4: 2.75–3 h, 3.5–4.5 h, and 5–6 h (for q6h) or 7–8 h (for q8h) after the start of the IV infusion

^*a*^ Subject PK data from the studies listed here were added to those included in the previously reported separate aztreonam and avibactam population PK models (12).

^*b*^ BAT, best available treatment.

^*c*^ BSI, bloodstream infection.

^*d*^ cIAI, complicated intra-abdominal infection.

^*e*^ cUTI, complicated urinary tract infection.

^*f*^ ELD, extended loading dose.

^*g*^ IV, intravenous.

^*h*^ HAP, hospital-acquired pneumonia.

^*i*^ LD, loading dose.

^*j*^ MBL, metallo β-lactamase.

^*k*^ MD, maintenance dose.

^*l*^ NP, nosocomial pneumonia.

^*m*^ PK, pharmacokinetic.

^*n*^ q6h, every 6 h.

^*o*^ q8h, every 8 h.

^*p*^ VAP, ventilator-associated pneumonia.

All studies included in the analyses were carried out in accordance with relevant regulatory and international standards, and institutional ethical approval was obtained for all trial protocols and amendments; all subjects (or their designated representatives) provided written informed consent.

### Analysis design

The first stage of the analysis included updating the data set of the previous simultaneous population PK model for aztreonam and avibactam (14), which included phase 1 and phase 2a aztreonam-avibactam data and data from the ceftazidime-avibactam program, with two additional phase 1 studies and two phase 3 adult studies and re-estimating the model parameters with an updated simultaneous population PK model. The final model obtained was then used to derive steady-state exposure metrics based on *post hoc* parameter estimates for patients in the aztreonam-avibactam phase 3 trials (5, 6) by infection type (cIAI, cUTI, and HAP/VAP) and renal function categories. Monte Carlo simulations using the final model explored exposures and joint probability of target attainment (PTA) across various loading dose scenarios, evaluated dose regimen scenarios for patients with ESRD, and supported final dose selection based on predicted exposures and joint PTA across patient populations. Given the importance of rapid PK/PD target attainment for antimicrobial efficacy, these simulations evaluated the first dosing interval and steady state. In addition, the final model was used to assess joint PTA at steady state for two proposed ceftazidime-avibactam + aztreonam dose regimens.

### Modeling software

The population PK modeling analyses were conducted using NONMEM, version 7.4.3 (ICON Development Solutions, Hanover, MD) and PsN version 5.3.0 (18, 19). R version 4.1.3 (https://www.r-project.org/) was used for data manipulation, exploratory analysis, and creation of tables and graphs. The preferred estimation method was first-order conditional estimation with interaction (20).

### Population pharmacokinetic model update

Plasma concentrations over time data for both aztreonam and avibactam were described by a two-compartment disposition model with zero-order infusion and first-order elimination processes in the simultaneous model. In prior modeling, body weight and baseline creatinine clearance (CrCL) normalized to body surface area (nCrCL) were included as structural covariates for aztreonam and avibactam (12). In addition, previously identified covariates for avibactam related to renal impairment, ESRD and dialysis on clearance (CL), and a phase 2 cIAI study effect on CL and central volume of distribution (Vc) were included in the base model (14, 21). Inter-individual variability (IIV) was included on aztreonam and avibactam CL and Vc, and on avibactam peripheral volume of distribution (Vp) and inter-compartmental CL (Q), using multiplicative exponential random effects based on the prior simultaneous model (14). Correlations between aztreonam and avibactam were linked via covariance terms of IIVs on CL and Vc.

The base PK model with the supported IIV terms was used as the starting point for the covariate model development. The covariate model building was performed using the stepwise covariate model (SCM) building procedure (22). The covariate search was not done in one forward and one backward, step as is the default in SCM. Instead, to make the default SCM more run time-efficient (run-time for most tested covariate models was >20 h), an initial univariate step was performed to identify the most relevant covariates, followed by two forward/backward searches (23). Goodness-of-fit of different models to the data was evaluated using the following criteria: change in the objective function value (OFV), visual inspection of various diagnostic plots, and precision of the parameter estimates. Prediction-corrected visual predictive checks (pcVPCs) were performed for the final model.

### Predicted exposures for phase 3 trial patients

The final simultaneous model was used to simulate steady-state PK profiles of aztreonam and avibactam for all patients in the phase 3 trials with available plasma concentration data (5, 6), using each patient’s individual *post hoc* PK parameter estimates and MD.

As time-varying nCrCL was included in the population PK model, simulations were based on the observed nCrCL taken closest to the last PK sampling day (days 4 ± 1). CrCL values (in mL/min) associated with these nCrCL values were used for categorizing patients into renal function groups as follows: ARC >150 mL/min; normal renal function >80 to ≤150 mL/min; mild renal impairment >50 to ≤80 mL/min; moderate renal impairment >30 ≤ 50 mL/min; severe renal impairment >15 to ≤30 mL/min; and ESRD <15 mL/min. CrCL values for each subject were kept constant throughout the time course of the simulations. Simulated total plasma concentrations were adjusted to free concentrations using unbound fractions of 0.616 for aztreonam and 0.92 for avibactam. Free plasma concentrations were used to calculate %*f*T >MIC of 8 mg/L for aztreonam and %*f*T >C_T_ of 2.5 mg/L for avibactam (11, 12).

### Simulations to support simplified loading doses

In the phase 3 trials, aztreonam-avibactam dose regimens consisted of a LD (30 min infusion), followed immediately by an ELD (3 h infusion) and MD (3 h infusion) starting at the next dose interval (5, 6). The LD and ELD were combined into a single infusion bag, necessitating a change in infusion rate after 30 min. A proposed SLD for clinical practice (one constant-rate 3 h infusion, with total dose equivalent to the phase 3 LD + ELD regimens) was compared in simulations with the phase 3 LD + ELD and with no loading dose. SLD simulations used the final simultaneous aztreonam-avibactam model and a virtual population of 5,000 patients with cIAI, the most common infection type in the clinical aztreonam-avibactam studies (52% of patients) and with the most conservative exposure estimates. Simulated total aztreonam and avibactam plasma concentrations (individual predictions [IPRED] incorporating *η*’s) were used to calculate PK and PK/PD exposure metrics. Concentration-time profiles for the first dosing interval after loading doses and at steady state (day 5) were simulated with time steps of 0.1 h. Covariates were sampled from a multivariate normal density with covariance of weight, body surface area (BSA), and CrCL based on adult subjects (split by renal function) in the current population PK analysis data set. nCrCL was computed based on sampled CrCL and BSA. The sampled weight and CrCL were truncated to the range obtained from the available adult data.

Predicted aztreonam and avibactam exposure metrics in the first dosing interval (maximum plasma concentration [C_max_] and area under the plasma concentration-time curve from time zero to the end of the dosing interval [AUC_τ_]) and at steady state (C_max,ss_ and AUC_24,ss_) based on total concentrations were calculated. Joint PTA for the primary PK/PD target of 60% *f*T >MIC of 8 mg/L for aztreonam and 50% *f*T >C_T_ of 2.5 mg/L for avibactam, achieved simultaneously, were compared for SLD + MD, phase 3 LD + ELD + MD, and MD-only regimens.

### Simulations to select a dose regimen for ESRD

Aztreonam-avibactam phase 3 dose regimens, which were selected based on an earlier population PK model (12), included adjustments for moderate (estimated CrCL >30 to ≤50 mL/min) and severe renal impairment (>15 to ≤30 mL/min). Dose adjustments for ESRD (CrCL ≤15 mL/min) requiring hemodialysis were not evaluated, and such patients were not included in the phase 3 trials (5, 6). To support the selection of a dose regimen recommendation for ESRD for clinical practice, the updated model was used to evaluate reduced SLD and/or MD doses and extended MD dosing intervals (every 8 h [q8h] and every 12 h [q12h]) in simulated cIAI patients with ESRD. Several dose regimen scenarios (administered after the hemodialysis session on hemodialysis days), maintaining the 3:1 ratio of aztreonam to avibactam, were explored, and predicted exposures and joint PTA in the first dose interval and at steady state were compared across scenarios and to other renal function groups.

### Simulations to confirm final dose regimens

Following the selection of SLDs and a dose regimen for ESRD (simulations done in patients with cIAI), the updated model was used to simulate concentration-time profiles for patients with cIAI, nosocomial pneumonia (NP; i.e., HAP and VAP), or cUTI, and to predict joint PTA by renal function group for the final selected dose regimens, including the SLD and dose regimen for ESRD; ARC (CrCL >150 mL/min) was evaluated using the dose regimen for normal renal function as administered to these patients in the phase 3 trials.

Simulations utilized the final simultaneous model with 5,000 patients per renal function category and covariate sampling as described above and were performed for different infection types (cIAI, NP [including HAP and VAP], and cUTI) with the final selected dose regimens.

### Simulations of ceftazidime-avibactam + aztreonam dose regimens

To assess the adequacy of PK/PD target attainment for the use of ceftazidime-avibactam + aztreonam combinations, the final model was used to simulate exposures and calculate joint PTA at steady state (5,000 patients/simulation) for two ceftazidime-avibactam + aztreonam dose regimens proposed by IDSA (17) in patients with cIAI with normal renal function (CrCL 80 to ≤150 mL/min).

## RESULTS

### Observed data

For aztreonam, 431 participants (325 males and 106 females) with 4,914 aztreonam PK plasma samples were included in the population PK analysis. For avibactam, 2,635 participants (1,618 males and 1,017 females) with 18,222 avibactam plasma samples were included. Baseline characteristics of subjects included in the population PK analysis are summarized in Tables S1 and S2.

### Final population PK model

The final population PK model was a two-compartment model for both aztreonam and avibactam with zero-order infusion and first-order elimination. PK parameter estimates for the base and final models are shown in Table S3. Body weight (normalized to 70 kg) was a structural covariate on both aztreonam and avibactam CL, Vc, Vp, and Q using allometric scaling with fixed exponents of 0.75 on CL and Q, and 1 on Vc and Vp. Time-varying nCrCL effects on aztreonam and avibactam CL were described by both linear (nCrCL *≥*80 mL/min/1.73 m^2^) and power (nCrCL <80 mL/min/1.73 m^2^) relationships for subjects not on dialysis.

Covariate effects of ESRD on avibactam CL, a separate avibactam CL for dialysis patients, and a separate avibactam CL and Vc for phase 2 patients with cIAI identified in prior modeling were retained. Acute physiologic assessment and chronic health evaluation (APACHE) II score on avibactam CL, also identified in prior models, remained significant after testing. Other population effects included cUTI effect on avibactam CL and Vc; cIAI effect on aztreonam and avibactam CL; same cIAI and NP (HAP/VAP) effect on aztreonam and avibactam Vc; Chinese race on avibactam Vc; an OMEGA block for variances of aztreonam CL and Vc and avibactam CL and Vc; a covariance term between the avibactam IIVs in Q and Vp; and separate residual error models for aztreonam and avibactam and study phases. Concentration data for both aztreonam and avibactam across studies were adequately described by the final model (Fig. S1 and S2). There were no trends for conditional weighted residuals (CWRES) versus time after first dose and population prediction (PRED) plots stratified by study or study phase. pcVPCs stratified by infection type versus time after dose and by baseline CrCL versus time after dose are shown in Fig. S3 to S6.

### Predicted exposures for phase 3 trial patients

Predicted steady-state aztreonam and avibactam exposures for phase 3 patients are summarized by renal function group in Table 2 and by infection type in Table 3. Patients with ARC had slightly lower aztreonam and avibactam exposures (AUC_24,ss_), whereas patients with moderate renal impairment had slightly higher exposures compared with other renal function groups (Table 2). Patients with NP (HAP or VAP) had modestly higher aztreonam and avibactam exposures compared with patients with cIAI (Table 3). This was predominantly driven by the higher exposures in patients with HAP; exposures of both drugs were similar between patients with VAP and cIAI. There were no obvious differences in aztreonam and avibactam exposures correlating with obesity (defined as body mass index *≥*30 kg/m^2^) and non-obesity, China and non-China (rest of world), or organic anion transporter (OAT)1/OAT3 inhibitor comedications (data not shown).

**TABLE 2:** Aztreonam and avibactam exposures at steady state for phase 3 patients by renal function groups^*a*^^,^^*b*^^,*c*,*d*,*e*,*f*^

	Aztreonam			Avibactam		
	N	C_max,ss_, mg/L	AUC_24,ss_, mg.h/L	N	C_max,ss_, mg/L	AUC_24,ss_, mg.h/L
ARC						
Mean (SD)	45	51.61 (27.1)	800.67 (527.69)	46	11.24 (6.93)	168.21 (123.12)
Median (range)	45	44.56 (15.53–194.22)	659.3 (70.45–3,779.55)	46	9.13 (3.05–44.79)	135.86 (13.68–805.91)
GM (%CV)	45	47.34 (41.09)	703.23 (55.92)	46	9.97 (49.04)	143.08 (62.08)
Normal renal function						
Mean (SD)	127	57.64 (18.7)	900.85 (338.36)	127	11.83 (4.16)	175.47 (68.63)
Median (range)	127	55.38 (6.47–135.02)	845.81 (91.38–2,263.32)	127	11.43 (1.04–29.94)	164.65 (15.77–462.7)
GM (%CV)	127	54.16 (40.83)	832.76 (45.83)	127	11.01 (44.86)	161.42 (47.5)
Mild renal impairment						
Mean (SD)	62	73.04 (23.19)	1,203.43 (423.1)	63	15.36 (5.95)	243.07 (105.74)
Median (range)	62	71.78 (17.3–153.66)	1,139.28 (267.16–2,541.53)	63	14.59 (3.2–39.01)	214.83 (52.14–737.23)
GM (%CV)	62	69.39 (34.48)	1,132 (37.6)	63	14.29 (41.85)	224.16 (42.73)
Moderate renal impairment						
Mean (SD)	27	78.07 (44.6)	1,396.22 (821.31)	27	19.22 (11.84)	341.8 (210.89)
Median (range)	27	63.94 (30.15–231.36)	1,093.57 (517.23–4,279.76)	27	14.21 (6.04–64.15)	281.4 (102.42–1,131.12)
GM (%CV)	27	68.45 (54.54)	1,220.4 (55)	27	16.82 (53.55)	299.05 (53.68)
Severe renal impairment						
Mean (SD)	8	52.26 (17.96)	928.97 (413.99)	8	16.38 (8.01)	318.04 (189.58)
Median (range)	8	47.94 (36.79–90.61)	822.26 (588.53–1,839.74)	8	15.77 (8.34–33.88)	310.45 (138.27–742.58)
GM (%CV)	8	50.01 (31.37)	865.86 (39.65)	8	14.98 (46.12)	279.63 (56.51)

^*a*^ CrCL values for renal function groups: ARC >150 mL/min; normal renal function >80 to ≤150 mL/min; mild renal impairment >50 to ≤80 mL/min; moderate renal impairment >30 to ≤50 mL/min; severe renal impairment >15 to ≤30 mL/min; ESRD <15 mL/min.

^*b*^ AUC24,ss, area under concentration-time curve over 24 hours at steady state.

^*c*^ Cmax,ss, maximum concentration at steady state.

^*d*^ %CV, percentage coefficient of variation.

^*e*^ GM, geometric mean.

^*f*^ SD, standard deviation.

**TABLE 3:** Aztreonam and avibactam exposures at steady state for phase 3 cIAI and NP patients^*a*^^,*b*,*c*,*d*,*e*,*f*,*g*,*h*,*i*^

	Aztreonam			Avibactam		
	N	C_max,ss_, mg/L	AUC_24,ss_, mg.h/L	N	C_max,ss_, mg/L	AUC_24,ss_, mg.h/L
cIAI						
Mean (SD)	191	57.54 (20.25)	916.81 (379.21)	192	12.36 (5.2)	190.59 (95.73)
Median (range)	191	54.86 (6.47–131.73)	843.44 (70.45–2541.53)	192	11.47 (1.04–39.01)	167.68 (13.68–737.23)
GM (%CV)	191	53.72 (41.6)	836.4 (49.52)	192	11.3 (47.96)	169.75 (54.97)
NP						
Mean (SD)	71	69.62 (29.54)	1154.35 (560.73)	72	15.21 (7.36)	244.85 (139.11)
Median (range)	71	63.94 (29.22–194.22)	1010.12 (443.64–3779.55)	72	13.42 (5.32–44.79)	208.86 (80.58–805.91)
GM (%CV)	71	64.61 (39.44)	1052.4(43.97)	72	13.86 (44.23)	217.41 (49.77)
HAP						
Mean (SD)	38	78.9 (34.41)	1339.16 (648.55)	39	17.7 (8.65)	292.18 (165.81)
Median (range)	38	74.15 (29.22–194.22)	1181.68 (443.64–3779.55)	39	15.23 (5.32–44.79)	231.59 (80.58–805.91)
GM (%CV)	38	72.69 (42.3)	1216.9 (45.79)	39	15.97 (47.83)	256.38 (54.31)
VAP						
Mean (SD)	33	58.94 (17.88)	941.53 (337.91)	33	12.28 (3.86)	188.91 (65.82)
Median (range)	33	56.37 (32.17–104.49)	904.08 (466.53–2133.71)	33	11.95 (6.12–21.17)	183 (88.09–412.88)
GM (%CV)	33	56.42 (30.78)	890.37 (34.55)	33	11.71 (32.09)	178.91 (34.39)

^*a*^ AUC24,ss, area under concentration-time curve over 24 hours at steady state.

^*b*^ cIAI, complicated intra-abdominal infection.

^*c*^ Cmax,ss, maximum concentration at steady state.

^*d*^ %CV, percentage coefficient of variation.

^*e*^ GM,geometric mean.

^*f*^ HAP, hospital-acquired pneumonia.

^*g*^ NP, nosocomial pneumonia.

^*h*^ SD, standard deviation.

^*i*^ VAP, ventilator-associated pneumonia.

### Simulations to support simplified loading doses

The proposed SLD for clinical practice (one constant-rate IV infusion over 3 h, with total dose equivalent to the phase 3 LD + ELD regimens) was compared with the phase 3 regimen and no LD using the final simultaneous population PK in patients with cIAI (Table 4; Tables S4 and S5). Comparing exposures of aztreonam and avibactam in the first dosing interval across renal function categories, SLD gave 9%–19% higher C_max_ compared with phase 3 LD + ELD. For the scenario without LD, aztreonam and avibactam C_max_ values were 41%–89% lower compared with LD + ELD (Tables S4 and S5). As the total doses administered were not different, AUC_τ_ values were equivalent for SLD and LD + ELD regimens. Exposure parameters at steady state (C_max,ss_ and AUC_24,ss_) were unaffected by loading dose scenarios.

**TABLE 4:** Joint PTA for simulated adult patients with cIAI across renal function groups for aztreonam-avibactam LD + ELD, SLD, and MD-only scenarios^*a*^^,*b*,*c*,*d*,*e*,*f*,*g*,*h*,*i*,*j*,*k*,*l*^

Renal function category	Dose regimen scenario	Aztreonam-avibactam dose (infusion duration)	Joint PTA, %	
			First dosing interval	Steady state
ARC	LD + ELD + MD	LD 500/167 mg (30 min) + ELD 1,500/500 mg (3 h)	94.9	89.4
	SLD + MD	SLD 2,000/667 mg (3 h)	93.3	90.2
	MD only	MD 1,500/500 mg (3 h), q6h	79.2	90.2
Normal renal function	LD + ELD + MD	LD 500/167 mg (30 min) + ELD 1,500/500 mg (3 h)	97.7	96.8
	SLD + MD	SLD 2,000/667 mg (3 h)	97.0	96.7
	MD only	MD 1,500/500 mg (3 h), q6h	89.1	96.7
Mild renal impairment	LD + ELD + MD	LD 500/167 mg (30 min) + ELD 1,500/500 mg (3 h)	99.4	99.4
	SLD + MD	SLD 2,000/667 mg (3 h)	99.3	99.3
	MD only	MD 1,500/500 mg (3 h), q6h	96.3	99.3
Moderate renal impairment	LD + ELD + MD	LD 500/167 mg (30 min) + ELD 1,500/500 mg (3 h)	99.8	95.2
	SLD + MD	SLD 2,000/667 mg (3 h)	99.8	95.0
	MD only	MD 750/250 mg (3 h), q6h	73.4	95.0
Severe renal impairment	LD + ELD + MD	LD 675/225 mg (30 min) + ELD 675/225 mg (3 h)	98.6	90.4
	SLD + MD	SLD 1,350/450 mg (3 h)	97.9	91.2
	MD only	MD 675/225 mg (3 h), q8h	68.3	91.2

^*a*^ Joint PTA for the plasma exposure targets of 60%fT >8 mg/L for aztreonam and 50%fT >2.5 mg/L for avibactam, achieved simultaneously. CrCL values for renal function groups: ARC >150 mL/min; normal renal function >80 to ≤150 mL/min; mild renal impairment >50 to ≤80 mL/min; moderate renal impairment >30 to ≤50 mL/min; severe renal impairment >15 to ≤30 mL/min; ESRD <15 mL/m.

^*b*^ ARC, augmented renal clearance.

^*c*^ cIAI, complicated intra-abdominal infection.

^*d*^ CrCL, creatinine clearance.

^*e*^ ELD, extended loading dose.

^*f*^ ESRD, end-stage renal disease.

^*g*^ LD, loading dose.

^*h*^ MD, maintenance dose.

^*i*^ PTA, probability of target attainment.

^*j*^ q6h, every 6 h.

^*k*^ q8h, every 8 h.

^*l*^ SLD, simplified loading dose.

Predicted joint PTA during the first dosing interval reached >90% in both the SLD and LD + ELD scenarios (Table 4). Without loading doses, joint PTA in the first dosing interval was <90% for all renal function groups except for mild renal impairment and was <80% for ARC and moderate or severe renal impairment.

### Simulations to select a dose regimen for ESRD

Among six simulated ESRD dose scenarios, aztreonam-avibactam SLD 1,000/334 mg and MD 675/225 mg q12h was associated with predicted aztreonam and avibactam C_max_ values in the first dose interval (Table S6) within the range of other renal function categories (Tables S4 and S5), with slightly higher AUC_τ_ values likely due to the longer dosing interval and lower CL. Joint PTA was 91.4% for this scenario (Table S6). At steady state, predicted aztreonam C_max,ss_ and AUC_24,ss_ for scenario 6 (Table S7) were also in the range of other renal function groups (Tables 2 and 3). Avibactam AUC_24,ss_ was greater than in other renal function groups, but the geometric mean was lower than the maximum values in phase 3 patients (Tables 2 and 3). Joint PTA was 89.1% (Table S7).

### Simulations to confirm the final dose regimens

Following the SLD and ESRD dose regimen simulations (all of which were done in patients with cIAI), further simulations using the final SLD and MD, and the selected ESRD dose regimen, were conducted for adults with cIAI, NP (HAP/VAP), and cUTI. Data for the first dose interval and steady state for simulated patients with cIAI are summarized in Tables S8 and S9, respectively. In the first dosing interval, patients with ARC had the lowest exposures (AUC_τ_) of both aztreonam and avibactam, and those with ESRD had the highest exposures (Table S8). At steady state, aztreonam exposures (AUC_24,ss_) were generally comparable across renal function groups (highest in mild renal impairment), whereas avibactam exposures were lowest in patients with ARC and highest in patients with ESRD.

Across renal function groups and for both the first dosing interval and at steady state, predicted median *ƒ*T >8 mg/L for aztreonam and *ƒ*T >2.5 mg/L for avibactam in simulated patients with cIAI were all >85% (Tables S8 and S9). Joint PTA was >90% both in the first dosing interval and at steady state (Table 5), except for patients with ESRD at steady state (89.1%). Aztreonam and avibactam exposures and median *ƒ*T > target concentrations for simulated NP and cUTI patients were generally higher than those of patients with cIAI across renal function groups (data not shown). Joint PTA in the first dose interval and at steady state for the approved dose regimens in simulated patients with NP and cUTI are shown in Tables S10 and S11, respectively. The approved regimens (Table 5) achieved >90% joint PTA at steady state across all renal function groups and infection types, other than ESRD in patients with cIAI, and ARC in patients with cUTI, for whom joint PTA was >89%.

**TABLE 5:** Joint PTA for simulated adult patients with cIAI across renal function groups for approved aztreonam-avibactam adult dose regimens^*a*^^,*b*,*c*,*d*,*e*,*f*,*g*,*h*,*i*,*j*,*k*^

Renal function category	First dosing interval		Steady state		
	Loading dose, mg	Joint PTA, %	Maintenance dose, mg	Frequency	Joint PTA, %
ARC	2,000/667	93.3	1,500/500	q6h	90.2
Normal renal function	2,000/667	97.0	1,500/500	q6h	96.7
Mild renal impairment	2,000/667	99.3	1,500/500	q6h	99.3
Moderate renal impairment	2,000/667	99.8	750/250	q6h	95.0
Severe renal impairment	1,350/450	97.9	675/225	q8h	91.2
ESRD	1,000/334	91.4	675/225	q12h	89.1

^*a*^ All aztreonam-avibactam doses given as 3 h infusions. Joint PTA for the plasma exposure targets of 60%fT >8 mg/L for aztreonam and 50%fT >2.5 mg/L for avibactam, achieved simultaneously. CrCL values for renal function groups: ARC >150 mL/min; normal renal function >80 to ≤150 mL/min; mild renal impairment >50 to ≤80 mL/min; moderate renal impairment >30 to ≤50 mL/min; severe renal impairment >15 to ≤30 mL/min; ESRD <15 mL/min.

^*b*^ ARC, augmented renal clearance.

^*c*^ cIAI, complicated intra-abdominal infection.

^*d*^ CrCL, creatinine clearance.

^*e*^ ESRD, end-stage renal disease.

^*f*^ LD, loading dose.

^*g*^ MD, maintenance dose.

^*h*^ PTA, probability of target attainment.

^*i*^ q6h, every 6 h.

^*j*^ q8h, every 8 h.

^*k*^ q12h every 12 h.

### Simulations of ceftazidime-avibactam + aztreonam dose regimens

In simulated patients with cIAI with CrCL 80 to ≤150 mL/min, ceftazidime-avibactam + aztreonam dose regimens resulted in similar (or higher) predicted geometric mean aztreonam C_max,ss_ and AUC_24,ss_ compared with the approved aztreonam-avibactam dose regimen (Table S9), but avibactam AUC_24,ss_ was approximately 25% lower, and joint PTA was <85% (Table 6). Failure to achieve joint PTA > 90% with both ceftazidime-avibactam + aztreonam dose regimens results from reduced avibactam exposures associated with the ceftazidime-avibactam + aztreonam dose regimens (total daily doses of 6 g ceftazidime, 1.5 g avibactam, and 6 g or 8 g aztreonam), and as such is independent of pathogen MIC.

**TABLE 6:** Predicted aztreonam and avibactam steady state exposures and joint PTA for proposed ceftazidime-avibactam + aztreonam-doses in simulated adults with cIAI (CrCL 80 to ≤150 mL/min)^*a*^^,*b*,*c*,*d*,*e*,*f*,*g*,*h*^

	Aztreonam		Avibactam		Joint PTA, %
Dose regimen (3 h infusions)	C_max,ss_, mg/L	AUC_24,ss_, mg.h/L	C_max,ss_, mg/L	AUC_24,ss_, mg.h/L	
Ceftazidime-avibactam 2 g/0.5 g q8h + aztreonam 2 g q6h	68.8 (40.0)	1,111 (44.9)	9.5 (49.8)	118 (48.6)	84.1
Ceftazidime-avibactam 2 g/0.5 g q8h + aztreonam 2 g q8h	64.7 (42.3)	845 (45.0)	9.7 (49.0)	120 (48.6)	84.3

^*a*^ Values are geometric mean (geometric %CV) for C_max_ and AUC. Joint PTA for the plasma exposure targets of 60%fT >8 mg/L for aztreonam and 50%fT >2.5 mg/L for avibactam, achieved simultaneously.

^*b*^ AUC24,ss, area under concentration-time curve over 24 hours at steady state.

^*c*^ cIAI, complicated intra-abdominal infection.

^*d*^ Cmax,ss, maximum concentration at steady state.

^*e*^ %CV, percentage coefficient of variation.

^*f*^ PTA, probability of target attainment.

^*g*^ q6h, every 6 h.

^*h*^ q8h, every 8 h.

## DISCUSSION

Aztreonam-avibactam offers a novel approach to addressing the problem of MBL-producing pathogens, and the completed adult clinical trial program has demonstrated the safety and clinical effectiveness of the combination (4–6). Development of aztreonam-avibactam has leveraged prior knowledge of aztreonam monotherapy (24–27) and avibactam population PK models developed for ceftazidime-avibactam (28, 29), as well as preclinical and *in vitro* microbiological data (13, 30–32). Approval of aztreonam-avibactam in adults in Europe (7) includes the final dose regimens outlined here. Aztreonam-avibactam has also recently been approved by the FDA (33) with regulatory review underway in other regions. Phase 3 dose regimen selection for aztreonam-avibactam was guided by existing data and dosing recommendations for aztreonam monotherapy (24, 25), *in vitro* MIC distributions for aztreonam and avibactam in combination against target pathogens (34–38), and an iterative series of population PK analyses and PTA simulations evaluating various dosing scenarios (12).

The current analyses used patient PK data from the completed aztreonam-avibactam adult phase 2a and phase 3 trials (4–6) to update a PK model that was used to estimate *post hoc* PK parameters and confirm exposures in phase 3 patients across infection types and renal function categories and explore (by simulations) SLD and ESRD dose regimens for clinical practice. The cIAI patient population was used for these simulations as most patients in the phase 2a and 3 trials had cIAI, and it is also the most conservative approach, since exposures of both aztreonam and avibactam were lowest in cIAI relative to other infection types.

The current analysis represents a significant increase in the number of plasma concentrations available for the characterization of aztreonam in patients with serious infections relative to previous modelling iterations (12, 14). Avibactam data from the ceftazidime-avibactam program (28, 29) were incorporated into the earlier model to inform the PK (including renal impairment groups) and variability in adults with cIAI, HAP/VAP, and cUTI (12). Correlations between the variances of aztreonam and avibactam CL and Vc were high at 0.976 and 0.986, respectively, as expected for drugs that are both predominantly renally cleared. The PK properties of both drugs following IV infusion were adequately characterized in simultaneous modeling by two-compartment disposition models with first-order elimination. Per previous assessments, it was difficult to estimate IIVs for aztreonam on Q and Vp, possibly because the majority of aztreonam data were collected at steady state (4–6).

Simulations based on *post hoc* parameter estimates showed that the aztreonam-avibactam phase 3 dose regimens achieved broadly similar steady-state exposures across renal function groups and infection types. Patients with ARC had slightly lower aztreonam and avibactam exposures (AUC_24,ss_), and those with moderate renal impairment had slightly higher exposures compared with patients in other renal function groups; these differences do not warrant further dose adjustments.

The current analyses underline the previously documented need for aztreonam-avibactam loading doses to support early PK/PD target attainment (12), an important consideration that can impact treatment outcomes for critically ill patients (10). The impact of loading doses on joint PTA in the first dose interval is particularly evident for patients with moderate or severe renal impairment or ARC (without loading dose, joint PTA in the first dose interval was <80% for ARC and moderate or severe renal impairment). Despite the importance of loading doses, the phase 3 LD + ELD regimens have the potential for dosing administration errors in clinical practice. To reduce this risk, simulations explored various SLD scenarios (administered as one constant-rate 3 h infusion with the same total dose as the phase 3 LD + ELD). In the SLD simulations, aztreonam and avibactam C_max_ values were <20% higher after SLD, whereas AUC_τ_ values were comparable; exposures at steady state were not impacted in any renal function group. These results supported the inclusion of SLD in the approved aztreonam-avibactam dose recommendations (7).

Dose regimens for patients with ESRD were assessed using a similar approach to that taken to select doses for other renal impairment groups, with the aim of matching exposures of aztreonam and avibactam to normal renal function while maintaining a 3:1 ratio of aztreonam to avibactam and achieving high joint PTA. A proposed aztreonam-avibactam regimen with 50% reduction in LD (1,000/334 mg) compared with normal renal function and the same MD as severe renal impairment (675/225 mg) with a longer dosing interval (q12h versus q8h) best achieved exposure goals and is included in the approved European dosing recommendations (7). Although predicted avibactam exposure at steady state for this ESRD regimen was higher than for other renal function groups, it did not exceed maximum exposures observed in the phase 3 trials (5, 6). Joint PTA at steady state for the selected ESRD dose regimen in simulated patients with cIAI was 89.1%, slightly below the target of >90%. This may be attributable to the lower exposure and longer dosing interval, resulting in fewer simulated patients achieving the aztreonam PK/PD target.

This analysis benefits from the use of the rich avibactam PK data set obtained largely from the ceftazidime-avibactam program. The simulations used a robust joint PTA target with a 50% *f*T >C_T_ of 2.5 mg/L for avibactam and 60% *f*T >MIC of 8 mg/L for aztreonam. In preclinical models, these exposure metrics correlated with the restoration of aztreonam efficacy against MBL-producing *Enterobacterales*; notably, the joint PTA target for aztreonam-avibactam is more stringent than that for the ceftazidime-avibactam combination, requiring 2.5-fold greater magnitude of C_T_ and a 1.2-fold greater duration of %*f*T >MIC (11). However, it should be noted that although the joint target used in these analyses supports the achievement of joint PTA in simulated patients for MICs up to 8 mg/L with the approved aztreonam-avibactam dose regimens, the European Committee on Antimicrobial Susceptibility Testing (EUCAST) has assigned “susceptible” and “resistant” MIC breakpoints against *Enterobacterales* of ≤4 and >4 mg/L respectively (tested using a fixed 4 mg/L concentration of avibactam) (39), reflecting a lack of clinical outcomes data for isolates with higher aztreonam-avibactam MICs. As dose regimens and PK/PD considerations are a key component of breakpoint determinations, the EUCAST MIC breakpoints for aztreonam-avibactam are not applicable to ceftazidime-avibactam + aztreonam dose regimens such as those proposed by IDSA (17). Our simulations of those regimens showed that joint PTA was <85% because of insufficient avibactam exposures, regardless of the aztreonam dose (2 g q6h or 2 g q8h). Hence, although there have been reports of positive clinical outcomes for ceftazidime-avibactam + aztreonam (40), this combination cannot be considered equivalent to aztreonam-avibactam because of the different antibiotic-to-inhibitor ratios for aztreonam-avibactam (3:1) and ceftazidime-avibactam (4:1) as well as the different plasma PK/PD targets for each combination,

Limitations of these analyses include the relatively small numbers of subjects and plasma PK observations for aztreonam (431 subjects, 4,914 plasma samples) compared with avibactam (2,635 subjects, 18,222 plasma samples). Moreover, the approved ESRD dose regimen is based on simulations only and has not been evaluated in clinical trials; the approved regimen requires patients to be receiving hemodialysis, with aztreonam-avibactam to be administered after dialysis. In summary, the current analyses showed that the aztreonam-avibactam phase 3 dose regimens achieved predicted exposures sufficient to achieve the robust joint PK/PD targets in adults with cIAI, NP (HAP/VAP), or cUTI with various degrees of renal function and supported recommendations for SLD and a dose regimen for ESRD patients. The SLD regimens (one constant-rate infusion over 3 h), which are included in the approved European product label (7), provide simplified administration while achieving the best match to the phase 3 dose regimen (LD/ELD) across infection types and renal function categories.

## Data Availability

Upon request, and subject to certain criteria, conditions, and exceptions, see https://www.pfizer.com/science/clinical-trials/trial-data-and-results for more information. Pfizer will provide access to individual de-identified participant data from Pfizer-sponsored global interventional clinical studies conducted for medicines, vaccines and medical devices (1) for indications that have been approved in the US and/or EU or (2) in programs that have been terminated (i.e., development for all indications has been discontinued). Pfizer will also consider requests for the protocol, data dictionary, and statistical analysis plan. Data may be requested from Pfizer trials 24 months after study completion. The de-identified participant data will be made available to researchers whose proposals meet the research criteria and other conditions, and for which an exception does not apply, via a secure portal. To gain access, data requestors must enter into a data access agreement with Pfizer.

## SUPPLEMENTAL MATERIAL

Supplemental material aac.01950-24-s0001.docx Tables S1 to S11; Fig. S1 to S6.

ASM does not own the copyrights to Supplemental Material that may be linked to, or accessed through, an article. The authors have granted ASM a non-exclusive, world-wide license to publish the Supplemental Material files. Please contact the corresponding author directly for reuse.
