# Supplementary material for: Population pharmacokinetic/pharmacodynamic modeling to optimize aztreonam-avibactam dose regimens for adult patients
Source: Antimicrob Agents Chemother. 2025 Jun 18;69(8):e01950-24. doi: 10.1128/aac.01950-24 (PMC12327849; doi:10.1128/aac.01950-24)
Supplement: Supplemental material — Tables S1 to S11; Fig. S1 to S6. [file aac.01950-24-s0001.docx]

# Supplemental appendix

Population pharmacokinetic/pharmacodynamic
modeling to optimize aztreonam-avibactam dose regimens for adult patients

**Rujia Xie,^1^ Halley Rogers,^2^ Joseph W. Chow,^3^ Elena Soto,^4^ Susan R. Raber^5^**

^1^Pfizer Ltd., Singapore; ^2^Pfizer Inc., New York, New York, USA; ^3^Pfizer Inc., Collegeville, Pennsylvania, USA; ^4^Pfizer Inc., Sandwich, Kent, UK; ^5^Pfizer Inc., La Jolla, California, USA

Table S1. Summary of number of subjects and PK samples and demographics in the aztreonam dataset

|  | **Healthy subjects** | **cIAI** | **HAP/VAP** | **cUTI** | **BSI** | **Overall** |
| --- | --- | --- | --- | --- | --- | --- |
| **Number of subjects** | 126 | 226 | 72 | 3 | 4 | 431 |
| **Number of plasma samples** | 3,108 | 1,386 | 379 | 18 | 23 | 4,914 |
| **Body weight, kg** | 77 (48–115) | 73 (37–130) | 70 (33–110) | 85 (48–107) | 78 (70–90) | 74 (33–130) |
| **BMI, kg/m^2^** | 24.8 (19.2–39.9) | 25.8 (14.2–58.6) | 24.2 (10.4–37.1) | 25 (19–40) | 27.0 (25.0–30.0) | 25.1 (10.4–58.6) |
| **Obesity** |  |  |  |  |  |  |
| BMI <30 kg/m^2^ | 115 (26.7) | 180 (41.8) | 62 (14.4) | 2 (0.5) | 3 (0.7) | 362 (84.0) |
| BMI ≥30 kg/m^2^ | 11 (2.6) | 46 (10.7) | 10 (2.3) | 1 (0.2) | 1 (0.2) | 69 (16.0) |
| **Age, years** | 34 (18–76) | 52 (18–86) | 65.5 (19–85) | 73 (32–83) | 66 (51–73) | 50 (18–86) |
| **Sex** |  |  |  |  |  |  |
| Males | 120 (27.8) | 148 (34.3) | 52 (12.1) | 3 (0.7) | 2 (0.5) | 325 (75.4) |
| Females | 6 (1.4) | 78 (18.1) | 20 (4.6) | 0 | 2 (0.5) | 106 (24.6) |
| **Baseline CrCL, mL/min** |  |  |  |  |  |  |
| >150 (*n* = 46)  ARC |  | 170.6 (150.3–349.6) | 178.7 (154.0–320.4) |  |  | 176.7 (150.3–349.6) |
| 80 to *≤*150 (*n* = 244) Normal renal function | 126.2 (81.7–231.6) | 115.7 (80.6–185.7) | 113.3 (80.3–148.0) | 134.2 | 92.9 | 117.2 (80.3–231.6) |
| 50 to *≤*80 (*n* = 74) Mild renal impairment | 62.3 (58.1–79.4) | 63.3 (50.5–80.0) | 60.6 (52.5–76.7) | 54.8 | 66.4 (64.7–68.1) | 62.9 (50.5–80.0) |
| 30 to *≤*50 (*n* = 38) Moderate renal impairment | 32.8 (31.9–48.7) | 41.6 (31.8–50.0) | 37.2 (30.3–49.0) |  | 49.2 | 38.6 (30.3–50.0) |
| 15 to *≤*30 (*n* = 17) Severe renal impairment | 18.9 (16.5–27.5) | 25.0 (20.0–28.9) | 19.8 (18.9–27.9) | 15.4 |  | 20.0 (15.4–28.9) |
| *≤*15 (*n* = 12) ESRD | 6.6 (4.6–14.8) |  |  |  |  | 6.6 (4.6–14.8) |
| **Race** |  |  |  |  |  |  |
| White | 56 (13.0) | 139 (32.3) | 41 (9.5) | 1 (0.2) | 2 (0.46) | 239 (55.5) |
| Black | 12 (2.8) | 1 (0.2) | 0 | 0 | 0 | 13 (3.0) |
| Asian | 6 (1.4) | 18 (4.2) | 9 (2.1) | 1 (0.2) | 2 (0.46) | 36 (8.4) |
| Native American | 0 | 7 (1.6) | 0 | 1 (0.2) | 0 | 8 (1.9) |
| Chinese | 8 (1.9) | 56 (13.0) | 20 (4.6) | 0 | 0 | 84 (19.5) |
| Other | 2 (0.5) | 3 (0.7) | 1 (0.2) | 0 | 0 | 6 (1.4) |
| Missing | 42 (9.7) | 2 (0.5) | 1 (0.2) | 0 | 0 | 45 (10.4) |

Continuous variables are presented as median (range) and categorical data are expressed as n (%), with the total numbers of subjects as denominator.

ARC, augmented renal clearance; BMI, body mass index; BSI, bloodstream infection; cIAI, complicated intra-abdominal infection; CrCL, creatinine clearance;
cUTI, complicated urinary tract infection; ESRD, end-stage renal disease HAP, hospital-acquired pneumonia; PK, pharmacokinetic; VAP, ventilator-associated pneumonia.

Table S2. Summary of number of subjects and PK samples and demographics in the avibactam dataset

|  | **Healthy subjects** | **cIAI** | **HAP/VAP** | **cUTI** | **BSI** | **Overall** |
| --- | --- | --- | --- | --- | --- | --- |
| **Number of subjects** | 430 | 1,012 | 482 | 707 | 4 | 2,635 |
| **Number of plasma samples** | 10,603 | 3,835 | 1,593 | 2,168 | 23 | 18,222 |
| **Body weight, kg** | 76 (44–171) | 70 (37–159) | 66 (33–190) | 73 (28–142) | 78 (70–90) | 70 (28–190) |
| **BMI, kg/m^2^** | 24.9 (17.5–62.5) | 24.5 (14.2–58.6) | 23.4 (10.4–60.0) | 26.0 (14.3–56.1) | 27.0  (25.0–30.0) | 24.7 (10.4–62.5) |
| **Obesity** |  |  |  |  |  |  |
| BMI <30 kg/m^2^ | 376 (14.3) | 851 (32.3) | 425 (16.1) | 527 (20.0) | 3 (0.1) | 2,182 (82.8) |
| BMI ≥30 kg/m^2^ | 54 (2.1) | 161 (6.1) | 57 (2.2) | 180 (6.8) | 1 (0.04) | 453 (17.2) |
| **Age, years** | 32 (18–78) | 50 (18–87) | 66 (18–89) | 57 (18–89) | 66 (51–73) | 52 (18–89) |
| **Sex** |  |  |  |  |  |  |
| Males | 352 (13.4) | 641 (24.3) | 361 (13.7) | 262 (9.9) | 2 (0.1) | 1618 (61.4) |
| Females | 78 (3.0) | 371 (14.1) | 121 (4.6) | 445 (16.9) | 2 (0.1) | 1017 (38.6) |
| **Baseline CrCL, mL/min** |  |  |  |  |  |  |
| >150 (*n* = 215) ARC | 212.5 (141.7–305.8) | 172.9 (150.3–349.6) | 191.3 (150.3–739.0) | 166.4 (150.2–310.5) |  | 177.7  (141.7–739.0) |
| 80 to *≤*150 (*n* = 1,434)  Normal renal function | 123.2 (80.6–384.1) | 110.5 (80.2–219.1) | 107.3 (80.1–149.4) | 107.1 (80.5–289.6) | 92.9 | 112.3 (80.1–384.1) |
| 50 to *≤*80 (*n* = 741)  Mild renal impairment | 71.5 (56.2–79.4) | 63.9  (50.5–80.0) | 62.1 (50.1–79.9) | 65.3 (50.2–80.0) | 66.4 (64.7–68.1) | 64.0  (50.1–80.0) |
| 30 to *≤*50 (*n* = 202)  Moderate renal impairment | 37.54 (32.3–48.1) | 39.0  (30.5–50.0) | 39.7 (30.3–49.7) | 41.4  (30.5–49.8) | 49.2 | 40.1 (30.3–50.0) |
| 15 to *≤*30 (*n* = 40)  Severe renal impairment | 22.2 (15.7–29.8) | 25.0 (20.0–28.9) | 24.7 (16.2–29.7) | 25.3 (15.4–28.7) |  | 23.8  (15.4–29.8) |
| <15 (*n* = 3)  ESRD *≤*15 | 13.8 |  |  | 12.6 (11.0–14.2) |  | 13.8  (11.0–14.2) |
| **Race** |  |  |  |  |  |  |
| White | 278 (10.6) | 584 (22.2) | 211 (8.0) | 583 (22.1) | 2 (0.08) | 1,658 (62.9) |
| Black | 78 (3.0) | 7 (0.3) | 2 (0.1) | 3 (0.1) | 0 | 90 (3.4) |
| Asian | 19 (0.7) | 190 (7.2) | 91 (3.5) | 22 (0.8) | 2 (0.08) | 324 (12.3) |
| Native American | 0 | 1 (0.04) | 13 (0.5) | 2 (0.08) | 0 | 16 (0.6) |
| Native Hawaiian | 0 | 0 | 1 (0.04) | 0 | 0 | 1 (0.04) |
| Japanese | 13 (0.5) | 0 | 13 (0.5) | 32 (1.2) | 0 | 58 (2.2) |
| Chinese | 20 (0.8) | 176 (6.7) | 156 (5.9) | 6 (0.2) | 0 | 358 (13.6) |
| Other | 21 (0.8) | 40 (1.5) | 7 (0.3) | 59 (2.2) | 0 | 127 (4.8) |
| Missing | 0 | 2 (0.1) | 1 (0.04) | 0 | 0 | 3 (0.1) |

Continuous variables are presented as median (range) and categorical data are expressed as n (%), with the total numbers of subjects as denominator.

ARC, augmented renal clearance; BMI, body mass index; BSI, bloodstream infection; cIAI, complicated intra-abdominal infection; CrCL, creatinine clearance; cUTI, complicated urinary tract infection; ESRD, end-stage renal disease HAP, hospital-acquired pneumonia; PK, pharmacokinetic; VAP, ventilator-associated pneumonia.

Table S3. **Parameter estimates for the base and final simultaneous aztreonam-avibactam population PK models**

|  | **Base model (run 1)** | | | **Final model (run 46)** | | |
| --- | --- | --- | --- | --- | --- | --- |
|  | **Estimate** | **RSE %** | **Shrinkage %** | **Estimate** | **RSE %** | **Shrinkage %** |
| CL_ATM (*θ_1_*) [L/h] | 5.58 | 2.81 |  | 5.00 | 2.37 |  |
| Vc_ATM (*θ_2_*) [L] | 11.7 | 3.24 |  | 7.01 | 3.34 |  |
| Q_ATM (*θ_3_*) [L/h] | 8.81 | 5.66 |  | 9.41 | 7.65 |  |
| Vp_ATM (*θ_4_*) [L] | 5.99 | 2.58 |  | 6.12 | 3.31 |  |
| CL_AVI (*θ_5_*) [L/h] | 10.8 | 1.45 |  | 10.3 | 1.61 |  |
| Vc_AVI (*θ_6_* [L] | 19.9 | 2.41 |  | 12.6 | 1.8 |  |
| Q_AVI (*θ_7_*) [L/h] | 5.34 | 3.82 |  | 4.82 | 3.54 |  |
| Vp_AVI (*θ_8_*) [L] | 7.27 | 2.26 |  | 6.95 | 1.75 |  |
| nCrCL on CL_ATM (*θ_9_*) | 0.477 | 7.58 |  | 0.502 | 7.11 |  |
| Slope nCrCL on CL_ATM (*θ_15_*) | 0.004 | 14.2 |  | 0.00383 | 14.6 |  |
| nCrCL on CL_AVI (*θ_10_*) | 1.05 | 4.57 |  | 1.06 | 4.61 |  |
| Slope nCrCL on CL_AVI (*θ_16_*) | 0.00302 | 10.3 |  | 0.00313 | 10.0 |  |
| ESRD on CL_AVI (*θ_11_*) | –0.914 | 2.23 |  | –0.923 | 1.95 |  |
| CL_AVI_DIAL (*θ_12_*) [L/h] | 21.2 | 10.5 |  | 17.9 | 11.2 |  |
| Study2002 on CL_AVI (*θ_13_*) | 0.995 | 15.6 |  | 0.89 | 16.7 |  |
| Study2002 on Vc_AVI (*θ_14_*) | 2.26 | 21.4 |  | 1.64 | 22.5 |  |
| cUTI on Vc_AVI (*θ_24_*) |  |  |  | 1.5 | 8.58 |  |
| cIAI/NP on Vc (*θ_25_*) |  |  |  | 0.931 | 6.37 |  |
| cUTI on CL_AVI (*θ_26_*) |  |  |  | 0.222 | 14.7 |  |
| cIAI on CL_AVI (*θ_27_*) |  |  |  | 0.115 | 17.7 |  |
| China on Vc_AVI (*θ_29_*) |  |  |  | –0.145 | 15.0 |  |
| APACHE II score on CL_AVI CL (*θ_30_*) |  |  |  | –0.118 | 12.3 |  |
| cIAI on CL_ATM (*θ_31_*) |  |  |  | 0.279 | 12.4 |  |
| IIV CL_ATM [CV%] | 44.1 | 26.6 | 17.18 | 40.2 | 25.8 | 15.52 |
| cov CL_ATM-Vc_ATM | 0.225 | 26.3 |  | 0.151 | 28.0 |  |
| IIV Vc_ATM [%CV] | 76.9 | 14.0 | 24.14 | 60.8 | 15.7 | 28.1 |
| cov CL_ATM-CL_AVI | 0.187 | 17.3 |  | 0.171 | 17.4 |  |
| cov CL_AVI-Vc_ATM | 0.212 | 18.3 |  | 0.173 | 19.0 |  |
| IIV CL_AVI [%CV] | 43.9 | 10.5 | 14.78 | 43.5 | 12.1 | 14.47 |
| cov CL_ATM-Vc_AVI | 0.269 | 20.0 |  | 0.182 | 21.4 |  |
| cov Vc_ATM-Vc_AVI | 0.661 | 12.2 |  | 0.391 | 14.5 |  |
| cov CL_AVI-Vc_AVI | 0.266 | 14.8 |  | 0.212 | 16.2 |  |
| IIV Vc_AVI [%CV] | 86.6 | 12.3 | 23.21 | 65.2 | 15.0 | 25.68 |
| IIV Q_AVI [%CV] | 35.7 | 21.6 | 70.22 | 31.4 | 23.0 | 72.12 |
| cov Q_AVI-Vp_AVI | 0.039 | 35.4 |  | 0.0309 | 37.7 |  |
| IIV Vp_AVI [%CV] | 18.0 | 30.4 | 71.43 | 17.7 | 29.7 | 71.56 |
| Additive RSV_ATM phase 1 (mg/L) (*θ_17_*) | 0.197 | 33.7 |  | 0.197 | 31.8 |  |
| Prop RSV_ATM (*θ_18_*) [%] | 12.5 | 7.24 |  | 12.5 | 7.18 |  |
| Additive RSV_AVI phase 1 (mg/L) (*θ_19_*) | 0.00615 | 11.5 |  | 0.00621 | 11.1 |  |
| Prop RSV_AVI (*θ_20_*) [%] | 20.0 | 6.21 |  | 20 | 6.21 |  |
| Prop RSV_ATM phase 2 (*θ_21_*) [%] | 22.3 | 15.6 |  | 22.4 | 16.3 |  |
| Prop RSV_ATM phase 3 (*θ_22_*) [%] | 40.0 | 7.62 |  | 40.3 | 7.48 |  |
| Prop RSV_ATM phase 2/3 (*θ_23_*) [%] | 51.7 | 2.92 |  | 53.3 | 3.05 |  |

APACHE, acute physiologic assessment and chronic health evaluation; ATM, aztreonam; AVI, avibactam;
cIAI, complicated intra-abdominal infection; CL, clearance; cov, covariance; cUTI, complicated urinary tract infection; %CV, percentage coefficient of variation; DIAL, dialysis; ESRD, end-stage renal disease;
IIV, inter-individual variability; nCrCL, body surface area-normalized creatinine clearance; NP, nosocomial pneumonia; PK, pharmacokinetic; Prop, proportion; Q, inter-compartmental clearance; RSE, relative standard error; RSV, residual variability; Vc, central volume of distribution; Vp, peripheral volume of distribution.

Table S4. Predicted aztreonam exposures for simulated adult patients with cIAI across renal function groups for aztreonam-avibactam LD + ELD, SLD, and MD only scenarios

|  |  | **First dosing interval** | | | | **Steady state** | |
| --- | --- | --- | --- | --- | --- | --- | --- |
|  | **Aztreonam-avibactam dose regimen (infusion duration)** | **C_max_, mg/L** | **C_max_ ratio** | **AUC**τ**, mg.h/L** | **AUC**_τ_ **ratio** | **C_max,ss_,**  **mg/L** | **AUC_24,ss_, mg.h/L** |
| **ARC** | LD 500/167 mg (30 min) + ELD 1,500/500 mg (3 h) + MD | 42.1 (42.4) |  | 171 (42.3) |  | 42.2 (42.3) | 660 (44.2) |
|  | SLD 2,000/667 mg (3 h) + MD | 49.2 (42.4) | 1.17 | 174 (41.7) | 1.02 | 42.4 (42.0) | 666 (44.0) |
|  | MD only 1500/500 mg (3 h), q6h | 36.9 (42.4) | 0.88 | 130 (41.7) | 0.76 | 42.4 (42.0) | 666 (44.0) |
| **Normal renal function** | LD 500/167 mg (30 min) + ELD 1,500/500 mg (3 h) + MD | 51.0 (42.8) |  | 208 (42.6) |  | 52.2 (42.7) | 843 (44.7) |
|  | SLD 2,000/667 mg (3 h) + MD | 58.2 (43.1) | 1.14 | 209 (42.3) | 1 | 51.9 (42.5) | 838 (44.6) |
|  | MD only 1,500/500 mg (3 h), q6h | 43.7 (43.1) | 0.86 | 156 (42.3) | 0.75 | 51.9 (42.5) | 838 (44.6) |
| **Mild renal impairment** | LD 500/167 mg (30 min) + ELD 1,500/500 mg (3 h) + MD | 60.9 (43.7) |  | 250 (43.4) |  | 65.1 (43.0) | 1,095 (44.8) |
|  | SLD 2000/667 mg (3 h) + MD | 68.8 (44.3) | 1.13 | 252 (43.0) | 1.01 | 65.0 (42.7) | 1,094 (44.5) |
|  | MD only 1500/500 mg (3 h), q6h | 51.6 (44.3) | 0.85 | 189 (43.0) | 0.76 | 65.0 (42.7) | 1,094 (44.5) |
| **Moderate renal impairment** | LD 500/167 mg (30 min) + ELD 1,500/500 mg (3 h) + MD | 69.5 (44.0) |  | 287 (43.6) |  | 39.8 (42.5) | 703 (44.4) |
|  | SLD 2,000/667 mg (3 h) + MD | 77.4 (45.1) | 1.11 | 291 (43.4) | 1.01 | 39.7 (42.6) | 699 (44.4) |
|  | MD only 750/250 mg (3 h), q6h | 29.0 (45.1) | 0.42 | 109 (43.4) | 0.38 | 39.7 (42.6) | 699 (44.4) |
| **Severe renal impairment** | LD 675/225 mg (30 min) + ELD 675/225 mg (3 h) + MD | 50.0 (46.4) |  | 291 (44.6) |  | 40.2 (44.3) | 647 (46.5) |
|  | SLD 1,350/450 mg (3 h) + MD | 59.5 (47.1) | 1.19 | 284 (44.0) | 0.98 | 40.3 (43.0) | 646 (44.9) |
|  | MD only 675/225 mg (3 h), q8h | 29.8 (47.1) | 0.59 | 142 (44.0) | 0.49 | 40.3 (43.0) | 646 (44.9) |
| **ESRD** | LD 675/225 mg (30 min) + ELD 675/225 mg (3 h) + MD | 58.4 (46.8) |  | 356 (45.6) |  | 55.8 (44.9) | 998 (47.3) |
|  | SLD 1,350/450 mg (3 h) + MD | 67.0 (49.6) | 1.15 | 346 (46.3) | 0.97 | 56.2 (44.9) | 1,008 (47.4) |
|  | MD only 675/225 mg (3 h), q8h | 33.5 (49.6) | 0.57 | 173 (46.3) | 0.49 | 56.2 (44.9) | 1,008 (47.4) |

Values are geometric mean (geometric %CV) for C_max_ and AUC. CrCL values for renal function groups: ARC >150 mL/min; normal renal function >80 to ≤150 mL/min;
mild renal impairment >50 to ≤80 mL/min; moderate renal impairment >30 to ≤50 mL/min; severe renal impairment >15 to ≤30 mL/min; ESRD <15 mL/min.

ARC, augmented renal clearance; AUC_24,ss_, area under concentration-time curve over 24 hours at steady state; AUC_τ_, area under concentration-time curve from time zero to the end of the dose interval; cIAI, complicated intra-abdominal infection; C_max_, maximum concentration; C_max,ss_, maximum concentration at steady state; CrCL, creatinine clearance; %CV, percentage coefficient of variation; ELD, extended loading dose; ESRD, end-stage renal disease; LD, loading dose; MD, maintenance dose; q6h, every 6 h; q8h, every 8 h; SLD, simplified loading dose.

Table S5. Predicted avibactam exposures for simulated adult patients with cIAI across renal function groups for aztreonam-avibactam LD + ELD, SLD and MD only scenarios

|  |  | **First dosing interval** | | | | **Steady state** | |
| --- | --- | --- | --- | --- | --- | --- | --- |
|  | **Aztreonam-avibactam dose regimen (infusion duration)** | **C_max_, mg/L** | **C_max_ ratio** | **AUC**τ**, mg.h/L** | **AUC**τ **ratio** | **C_max,ss_,**  **mg/L** | **AUC_24,ss_, mg.h/L** |
| **ARC** | LD 500/167 mg (30 min) + ELD 1500/500 mg (3 h) + MD | 8.43 (49.0) |  | 34.0 (48.8) |  | 8.39 (48.7) | 127 (48.1) |
|  | SLD 2000/667 mg (3 h) + MD | 10.0 (49.9) | 1.19 | 34.6 (48.1) | 1.02 | 8.45 (48.3) | 128 (47.9) |
|  | MD only 1500/500 mg (3 h), q6h | 7.53 (49.9) | 0.89 | 25.9 (48.1) | 0.76 | 8.45 (48.3) | 128 (47.9) |
| **Normal renal function** | LD 500/167 mg (30 min) + ELD 1500/500 mg (3 h) + MD | 10.2 (48.9) |  | 41.2 (48.7) |  | 10.3 (48.4) | 160 (47.9) |
|  | SLD 2000/667 mg (3 h) + MD | 11.8 (50.6) | 1.16 | 41.2 (48.7) | 1.00 | 10.2 (48.6) | 158 (48.3) |
|  | MD only 1500/500 mg (3 h), q6h | 8.84 (50.6) | 0.87 | 30.9 (48.7) | 0.75 | 10.2 (48.6) | 158 (48.3) |
| **Mild renal impairment** | LD 500/167 mg (30 min) + ELD 1500/500 mg (3 h) + MD | 12.8 (50.4) |  | 52.6 (49.9) |  | 13.8 (48.7) | 229 (48.7) |
|  | SLD 2000/667 mg (3 h) + MD | 14.7 (52.2) | 1.14 | 53.0 (49.6) | 1.01 | 13.8 (48.7) | 229 (48.8) |
|  | MD only 1500/500 mg (3 h), q6h | 11.0 (52.2) | 0.86 | 39.8 (49.6) | 0.76 | 13.8 (48.7) | 229 (48.8) |
| **Moderate renal impairment** | LD 500/167 mg (30 min) + ELD 1500/500 mg (3 h) + MD | 16.2 (52.6) |  | 67.6 (51.8) |  | 10.3 (48.6) | 190 (49.2) |
|  | SLD 2000/667 mg (3 h) + MD | 17.9 (54.7) | 1.1 | 68.3 (51.5) | 1.01 | 10.3 (48.2) | 188 (48.6) |
|  | MD only 750/250 mg (3 h), q6h | 6.71 (54.7) | 0.41 | 25.6 (51.5) | 0.38 | 10.3 (48.2) | 188 (48.6) |
| **Severe renal impairment** | LD 675/225 mg (30 min) + ELD 675/225 mg (3 h) + MD | 12.6 (56.0) |  | 77.0 (54.2) |  | 13.0 (51.9) | 237 (52.8) |
|  | SLD 1350/450 mg (3 h) + MD | 14.5 (59.3) | 1.15 | 74.8 (53.7) | 0.97 | 13.0 (50.0) | 237 (50.6) |
|  | MD only 675/225 mg (3 h), q8h | 7.25 (59.3) | 0.58 | 37.4 (53.7) | 0.49 | 13.0 (50.0) | 237 (50.6) |
| **ESRD** | LD 675/225 mg (30 min) + ELD 675/225 mg (3 h) + MD | 15.1 (59.3) |  | 97.5 (57.9) |  | 27.2 (56.0) | 571 (57.8) |
|  | SLD 1350/450 mg (3 h) + MD | 16.5 (63.7) | 1.09 | 93.9 (59.1) | 0.96 | 27.4 (56.4) | 576 (58.2) |
|  | MD only 675/225 mg (3 h), q8h | 8.25 (63.7) | 0.55 | 46.9 (59.1) | 0.48 | 27.3 (56.2) | 574 (57.9) |

Values are geometric mean (geometric %CV) for C_max_ and AUC. CrCL values for renal function groups: ARC >150 mL/min; normal renal function >80 to ≤150 mL/min; mild renal impairment >50 to ≤80 mL/min; moderate renal impairment >30 to ≤50 mL/min; severe renal impairment >15 to ≤30 mL/min; ESRD <15 mL/min.

ARC, augmented renal clearance; AUC_24,ss_, area under concentration-time curve over 24 hours at steady state; AUCτ, area under concentration-time curve from time zero to the end of the dose interval; C_max_, maximum concentration; C_max,ss_, maximum concentration at steady state; CrCL, creatinine clearance; ELD, extended loading dose; ESRD, end-stage renal disease; LD, loading dose; MD, maintenance dose; q6h, every 6 h; q8h, every 8 h; SLD, simplified loading dose.

Table S6. Summary of aztreonam and avibactam exposures, overall time above plasma PK/PD targets, and joint PTA in the first dose interval for ESRD dose regimen scenarios S1–S6 in simulated adult patients with cIAI

|  | **Aztreonam-avibactam dose (3-h infusions)** | | **Aztreonam** | | | **Avibactam** | | |  |
| --- | --- | --- | --- | --- | --- | --- | --- | --- | --- |
| **Dose regimen scenario** | **SLD** | **MD** | **C_max_, mg/L** | **AUCτ, mg.h/L** | **%*f*T >8 mg/L** | **C_max_, mg/L** | **AUCτ, mg.h/L** | **%*f*T >2.5 mg/L** | **Joint PTA, %** |
| **S1** | 1,000/334 mg | 500/167 mg q8h | 49.6 (49.6) | 256 (46.3) | 92.6 (75.3, 97.5) | 12.2 (63.7) | 69.7 (59.1) | 92.6  (77.8, 97.5) | 97.1 |
| **S2** | 1,000/334 mg | 450/150 mg q8h | 49.6 (49.6) | 256 (46.3) | 92.6 (75.3, 97.5) | 12.2 (63.7) | 69.7 (59.1) | 92.6 (77.8, 97.5) | 97.1 |
| **S3** | 1,000/334 mg | 375/125 mg q8h | 49.6 (49.6) | 256 (46.3) | 92.6 (75.3, 97.5) | 12.2 (63.7) | 69.7 (59.1) | 92.6 (77.8, 97.5) | 97.1 |
| **S4** | 1,350/450 mg | 675/225 mg q12h | 66.3 (49.3) | 450 (44.8) | 95.9 (71.1, 98.3) | 16.3 (63.0) | 135 (56.2) | 95.9 (89.3, 98.3) | 98.0 |
| **S5** | 675/225 mg | 675/225 mg q12h | 33.2 (49.3) | 225 (44.8) | 75.2 (14.0, 96.7) | 8.1 (63.0) | 67.4 (56.2) | 92.6 (24.8, 97.5) | 67.8 |
| **S6** | 1,000/334 mg | 675/225 mg q12h | 49.1 (49.3) | 333 (44.8) | 93.4 (52.1, 98.3) | 12.1 (63.0) | 100 (56.2) | 95.0 (85.1, 98.3) | 91.4 |

Values are geometric mean (geometric %CV) for C_max_ and AUC, and median (5th, 95th percentiles) for %*f*T based on 5,000 simulated patients per renal function category. Joint PTA for the plasma exposure targets of 60%*f*T >8 mg/L for aztreonam and 50%*f*T >2.5 mg/L for avibactam, achieved simultaneously.
AUC_τ_, area under concentration-time curve from time zero to the end of the dose interval; cIAI, complicated intra-abdominal infection; C_max_, maximum concentration; %CV, percentage coefficient of variation; ESRD, end-stage renal disease; MD, maintenance dose; PD, pharmacodynamic; PK, pharmacokinetic; PTA, probability of target attainment; q8h, every 8 h; q12h, every 12 h.

Table S7. Summary of aztreonam and avibactam exposures, overall time above plasma PK/PD targets, and joint PTA at steady state for ESRD dose regimens S1–S6 in simulated adult patients with cIAI

|  | **Aztreonam-avibactam dose (3-h infusions)** | | **Aztreonam** | | | **Avibactam** | | |  |
| --- | --- | --- | --- | --- | --- | --- | --- | --- | --- |
| **Dose regimen scenario** | **SLD** | **MD** | **C_max,ss_, mg/L** | **AUC_24,ss_, mg.h/L** | **%*f*T >8 mg/L** | **C_max,ss_, mg/L** | **AUC_24,ss_, mg.h/L** | **%*f*T >2.5 mg/L** | **Joint PTA, %** |
| **S1** | 1,000/334 mg | 500/167 mg q8h | 41.6 (44.9) | 746 (47.4) | 100 (64.2, 100) | 20.3 (56.4) | 428 (58.2) | 100 (100, 100) | 95.8 |
| **S2** | 1,000/334 mg | 450/150 mg q8h | 37.5 (45.0) | 672 (47.4) | 100 (53.1, 100) | 18.3 (56.5) | 385 (58.3) | 100 (100, 100) | 93.2 |
| **S3** | 1,000/334 mg | 375/125 mg q8h | 31.2 (45.0) | 560 (47.4) | 100 (28.4, 100) | 15.3 (56.6) | 321 (58.4) | 100 (100, 100) | 86.0 |
| **S4** | 1,350/450 mg | 675/225 mg q12h | 45.1 (44.7) | 661 (47.5) | 100 (46.3, 100) | 19.9 (55.6) | 380 (58.3) | 100 (100, 100) | 89.1 |
| **S5** | 675/225 mg | 675/225 mg q12h | 45.1 (44.7) | 661 (47.5) | 100 (46.3, 100) | 19.8 (55.4) | 377 (57.9) | 100 (100, 100) | 89.1 |
| **S6** | 1,000/334 mg | 675/225 mg q12h | 45.1 (44.7) | 661 (47.5) | 100 (46.3, 100) | 19.8 (55.5) | 378 (58.1) | 100 (100, 100) | 89.1 |

Values are geometric mean (geometric CV%) for C_max_ and AUC, and median (5th, 95th percentiles) for %*f*T based on 5,000 simulated patients per renal function category. Joint PTA for the plasma exposure targets of 60%*f*T >8 mg/L for aztreonam and 50%*f*T >2.5 mg/L for avibactam, achieved simultaneously.
AUC_24,ss_, area under concentration-time curve over 24 hours at steady state; cIAI, complicated intra-abdominal infection; C_max_, maximum concentration; C_max,ss_, maximum concentration at steady state; %CV, percentage coefficient of variation; ESRD, end-stage renal disease; MD, maintenance dose; PD, pharmacodynamic; PK, pharmacokinetic; PTA, probability of target attainment; q8h, every 8 h; q12h, every 12 h; SLD, simplified loading dose.

Table S8. Aztreonam and avibactam exposures and overall time above plasma PK/PD targets in the first dose interval in simulated adult patients with cIAI across renal function groups for approved aztreonam-avibactam dose regimens

| **Renal function group** | **Aztreonam-avibactam SLD (3-h infusions)** | **Aztreonam** | | | **Avibactam** | | |
| --- | --- | --- | --- | --- | --- | --- | --- |
|  |  | **C_max_, mg/L** | **AUCτ, mg.h/L** | **%*f*T >8 mg/L** | **C_max_, mg/L** | **AUCτ, mg.h/L** | **%*f*T >2.5 mg/L** |
| ARC | 2,000/667 mg | 49.2 (42.4) | 174 (41.7) | 90.2 (57.4, 96.7) | 10.0 (49.9) | 34.6 (48.1) | 85.2 (49.2, 96.7) |
| Normal renal function | 2,000/667 mg | 58.2 (43.1) | 209 (42.3) | 93.4 (68.9, 96.7) | 11.8 (50.6) | 41.2 (48.7) | 91.8 (59.0, 96.7) |
| Mild renal impairment | 2,000/667 mg | 68.8 (44.3) | 252 (43.0) | 93.4 (82.0, 98.4) | 14.7 (52.2) | 53.0 (49.6) | 93.4 (77.0, 98.4) |
| Moderate renal impairment | 2,000/667 mg | 77.4 (45.1) | 291 (43.4) | 95.1 (85.2, 98.4) | 17.9 (54.7) | 68.3 (51.5) | 95.1 (83.6, 98.4) |
| Severe renal impairment | 1,350/450 mg | 59.5 (47.1) | 284 (44.0) | 93.8 (74.1, 97.5) | 14.5 (59.3) | 74.8 (53.7) | 93.8 (82.7, 97.5) |
| ESRD | 1,000/334 mg | 49.1 (49.3) | 333 (44.8) | 93.4 (52.1, 98.3) | 12.1 (63.0) | 100 (56.2) | 95.0 (85.1, 98.3) |

Values are geometric mean (geometric %CV,) for C_max_ and AUC, and median (5th, 95th percentiles) for %*f*T based on 5,000 simulated patients per renal function category. CrCL values for renal function groups: ARC >150; normal renal function: >80 to ≤150; mild renal impairment >50 to ≤80; moderate renal impairment >30 ≤50; severe renal impairment >15 to ≤30; ESRD <15 mL/min.
ARC, augmented renal clearance; AUC_τ_, area under concentration-time curve from time zero to the end of the dose interval; cIAI, complicated intra-abdominal infection; C_max_, maximum concentration; CrCL, creatinine clearance; %CV, percentage coefficient of variation; ESRD, end-stage renal disease; PD, pharmacodynamic; PK, pharmacokinetic; SLD, simplified loading dose.

Table S9. Aztreonam and avibactam exposures and overall time above plasma PK/PD targets at steady state in simulated adult patients with cIAI across renal function groups for approved aztreonam-avibactam dose regimens

| **Renal function group** | **Aztreonam-avibactam MD (3-h infusions)** | **Aztreonam** | | | **Avibactam** | | |
| --- | --- | --- | --- | --- | --- | --- | --- |
|  |  | **C_max,ss_, mg/L** | **AUC_24,ss_, mg.h/L** | **%*f*T >8 mg/L** | **C_max,ss_, mg/L** | **AUC_24,ss_, mg.h/L** | **%*f*T >2.5 mg/L** |
| ARC | 1,500/500 mg q6h | 42.4 (42.0) | 666 (44.0) | 96.7 (54.1, 100) | 8.5 (48.3) | 128 (47.9) | 85.2 (44.3, 100) |
| Normal renal function | 1,500/500 mg q6h | 51.9 (42.5) | 838 (44.6) | 100 (67.2, 100) | 10.2 (48.6) | 158 (48.3) | 100 (57.4, 100) |
| Mild renal impairment | 1,500/500 mg q6h | 65.0 (42.7) | 1094 (44.5) | 100 (83.6, 100) | 13.8 (48.7) | 229 (48.8) | 100 (80.3, 100) |
| Moderate renal impairment | 750/250 mg q6h | 39.7 (42.6) | 699 (44.4) | 100 (60.7, 100) | 10.3 (48.2) | 188 (48.6) | 100 (80.3, 100) |
| Severe renal impairment | 675/225 mg q8h | 40.3 (43.0) | 646 (44.9) | 100 (49.4, 100) | 13.0 (50.0) | 237 (50.6) | 100 (100, 100) |
| ESRD | 675/225 mg q12h | 45.1 (44.7) | 661 (47.5) | 100 (46.3, 100) | 19.8 (55.5) | 378 (58.1) | 100 (100, 100) |

Values are geometric mean (geometric %CV) for C_max_ and AUC, and median (5th, 95th percentiles) for %*f*T based on 5,000 simulated patients per renal function category. CrCL values for renal function groups: ARC >150 mL/min; normal renal function >80 to ≤150 mL/min; mild renal impairment >50 to ≤80 mL/min; moderate renal impairment >30 to ≤50 mL/min; severe renal impairment >15 to ≤30 mL/min; ESRD <15 mL/min.
ARC, augmented renal clearance; AUC_24,ss_, area under concentration-time curve over 24 hours at steady state; cIAI, complicated intra-abdominal infection; C_max_, maximum concentration; C_max,ss_, maximum concentration at steady state; CrCL, creatinine clearance; %CV, percentage coefficient of variation; ESRD, end-stage renal disease;
MD, maintenance dose; PD, pharmacodynamic; PK, pharmacokinetic; q6h, every 6 h; q8h, every 8 h; q12h, every 12 h.

Table S10. Joint PTA for simulated adult patients with NP across renal function groups for the approved aztreonam-avibactam adult dose regimens

|  | **First dosing interval** | | **Steady state** | |
| --- | --- | --- | --- | --- |
|  | **Aztreonam-avibactam SLD (3-h infusions)** | **Joint PTA, %** | **Aztreonam-avibactam MD (3-h infusions)** | **Joint PTA, %** |
| ARC | 2,000/667 mg | 96.1 | 1,500/500 mg q6h | 95.9 |
| Normal renal function | 2,000/667 mg | 98.3 | 1,500/500 mg q6h | 98.4 |
| Mild renal impairment | 2,000/667 mg | 99.5 | 1,500/500 mg q6h | 99.9 |
| Moderate renal impairment | 2,000/667 mg | 99.9 | 750/250 mg q6h | 98.9 |
| Severe renal impairment | 1,350/450 mg | 99.2 | 675/225 mg q8h | 97.5 |
| ESRD | 1.000/334 mg | 96.3 | 675/225 mg q12h | 96.6 |

Joint PTA for the plasma exposure targets of 60%*f*T >8 mg/L for aztreonam and 50%*f*T >2.5 mg/L for avibactam, achieved simultaneously. CrCL values for renal function groups: ARC >150 mL/min; normal renal function >80 to ≤150 mL/min; mild renal impairment >50 to ≤80 mL/min; moderate renal impairment >30 ≤50 mL/min; severe renal impairment >15 to ≤30 mL/min; ESRD <15 mL/min.

ARC, augmented renal clearance; ATM, aztreonam; AVI, avibactam; CrCL, creatinine clearance;
cUTI, complicated urinary tract infection; ELD, extended loading dose; ESRD, end-stage renal disease;
MD, maintenance dose; PTA, probability of target attainment; q6h, every 6 h; q8h, every 8 h; q12h, every 12 h; SLD, simplified loading dose.

Table S11. Joint PTA for simulated adult patients with cUTI across renal function groups for the approved aztreonam-avibactam adult dose regimens

|  | **First dosing interval** | | **Steady state** | |
| --- | --- | --- | --- | --- |
|  | **Aztreonam-avibactam SLD (3-h infusions)** | **Joint PTA, %** | **Aztreonam-avibactam MD (3-h infusions)** | **Joint PTA, %** |
| ARC | 2,000/667 mg | 90.7 | 1,500/500 mg q6h | 89.5 |
| Normal renal function | 2,000/667 mg | 95.2 | 1,500/500 mg q6h | 95.8 |
| Mild renal impairment | 2,000/667 mg | 98.4 | 1,500/500 mg q6h | 99.3 |
| Moderate renal impairment | 2,000/667 mg | 99.6 | 750/250 mg q6h | 98.0 |
| Severe renal impairment | 1,350/450 mg | 97.9 | 675/225 mg q8h | 96.3 |
| ESRD | 1.000/334 mg | 95.2 | 675/225 mg q12h | 94.3 |

Joint PTA for the plasma exposure targets of 60%*f*T >8 mg/L for aztreonam and 50%*f*T >2.5 mg/L for avibactam, achieved simultaneously. CrCL values for renal function groups: ARC >150 mL/min; normal renal function >80 to ≤150 mL/min; mild renal impairment >50 to ≤80 mL/min; moderate renal impairment >30 ≤50 mL/min; severe renal impairment >15 to ≤30 mL/min; ESRD <15 mL/min.

ARC, augmented renal clearance; ATM, aztreonam; AVI, avibactam; CrCL, creatinine clearance; cUTI, complicated urinary tract infection; ELD, extended loading dose; ESRD, end-stage renal disease; MD, maintenance dose; PTA, probability of target attainment; q6h, every 6 h; q8h, every 8 h; q12h, every 12 h; SLD, simplified loading dose.

Figure S1. Basic goodness of fit for the final model for aztreonam


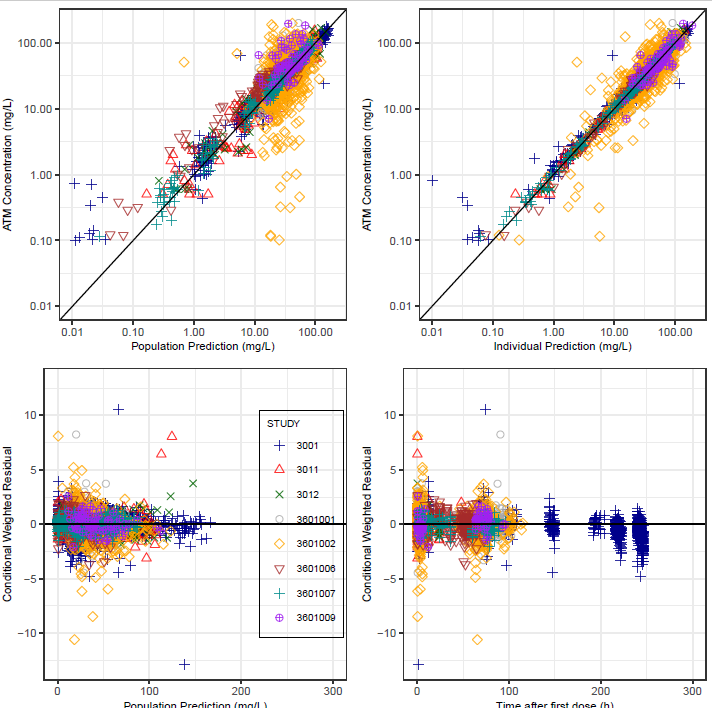


3601001 = Phase 2a REJUVENATE (4)
3011 = literature data (individual level) (40)
3001 = Phase 1 dose-finding study (41)
3012 = literature data (individual level) (42)
3601002 = Phase 3 REVISIT (5)
3601006 = Phase 1 renal impairment study (15)
3601007 = Phase 1 China PK Study (16)
3601009 = Phase 3 ASSEMBLE (6)
ATM, aztreonam.

Figure S2. Basic goodness of fit for the final model for avibactam


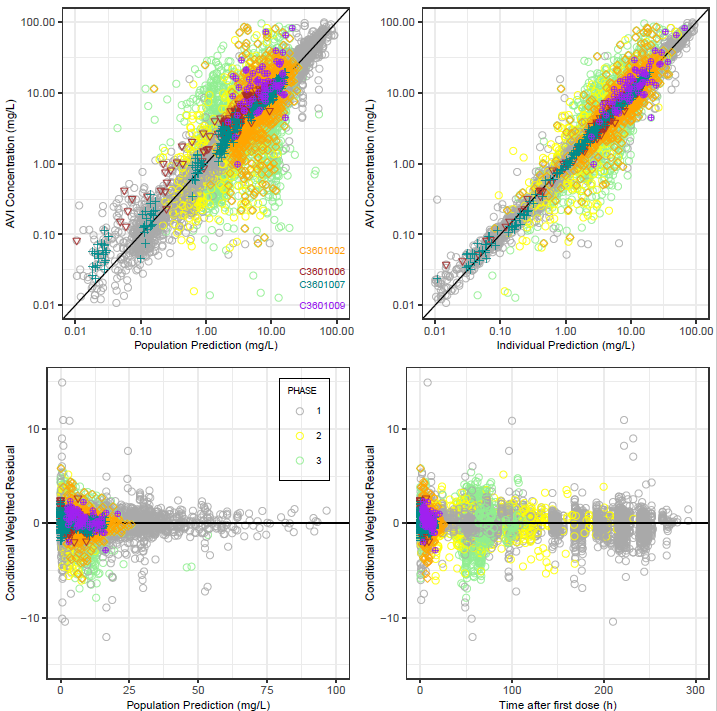


C3601002 = Phase 3 REVISIT (5)
C3601006 = Phase 1 renal impairment study (15)
C3601007 = Phase 1 China PK Study (16)
C3601009 = Phase 3 ASSEMBLE (6)
AVI, avibactam.

Figure S3. Prediction corrected visual predictive check stratified by infection type versus time after dose for the final model for aztreonam


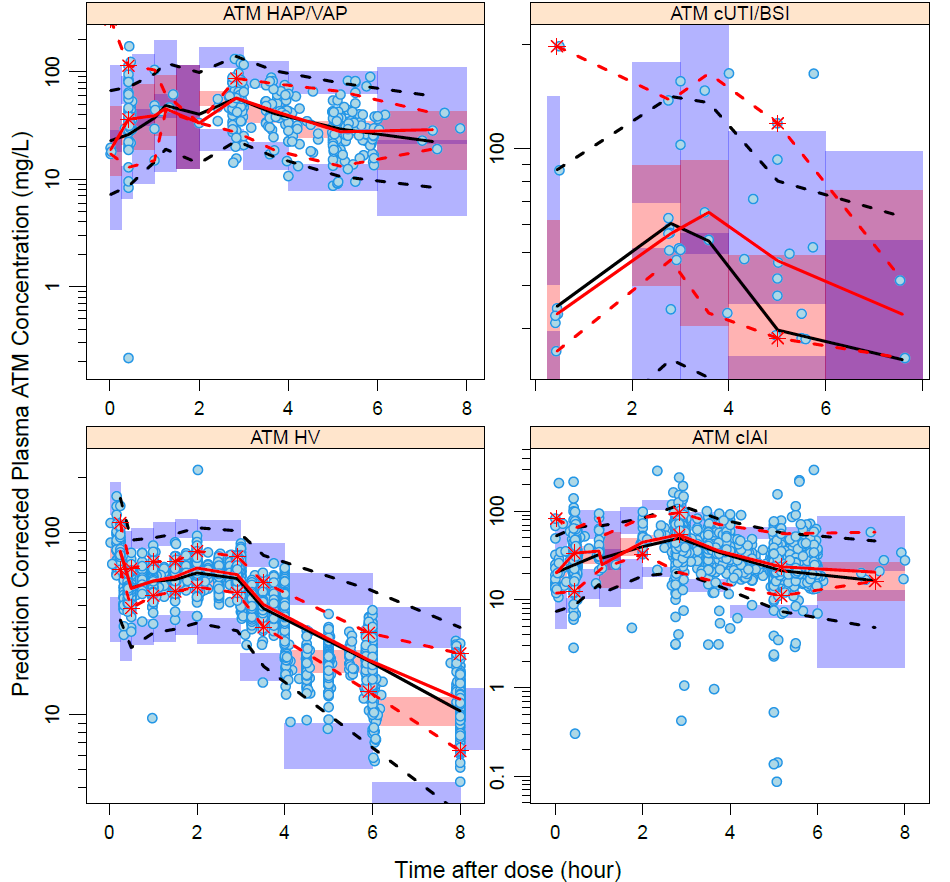


Red and black lines are the 5th, 50th (solid) and 95th percentile of observed and simulated data, respectively. Blue dots are observed data. Shadow areas are 95% confidence interval (CI) for the 5th, 50th (red) and 95th percentile prediction intervals based on simulated data. x-axis is truncated to 8 hours.

ATM = aztreonam; HV = healthy volunteer; cIAI = complicated intra-abdominal infection; cUTI = complicated urinary tract infection; BSI = bloodstream infection; HAP = hospital-acquired pneumonia; VAP = ventilator-associated pneumonia.

Figure S4. Prediction corrected visual predictive check stratified by infection type versus time after dose for the final model for avibactam


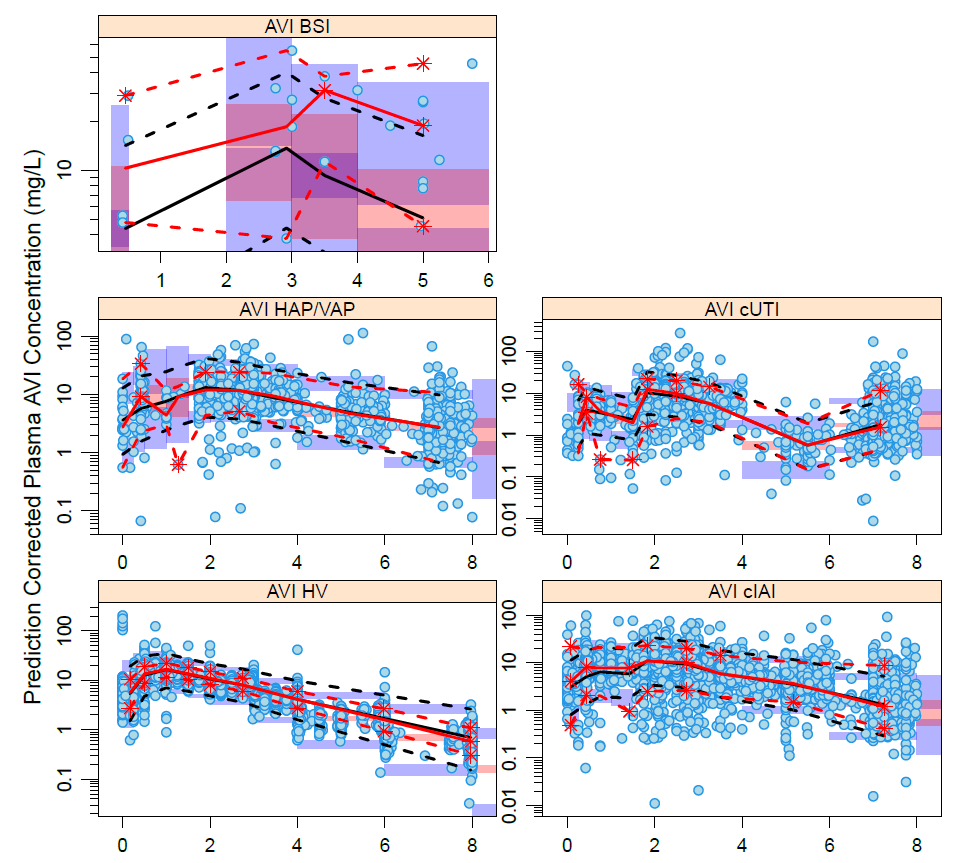


Red and black lines are the 5th, 50th (solid) and 95th percentile of observed and simulated data, respectively. Blue dots are observed data. Shadow areas are 95% confidence interval (CI) for the 5th, 50th (red) and 95th percentile prediction intervals based on simulated data. x-axis is truncated to 8 hours.

AVI = avibactam; HV = healthy volunteer; cIAI = complicated intra-abdominal infection; cUTI = complicated urinary tract infection; BSI = bloodstream infection; HAP = hospital-acquired pneumonia; VAP = ventilator-associated pneumonia.

Figure S5. Prediction corrected visual predictive check stratified by renal function category versus time after dose for the final model for aztreonam


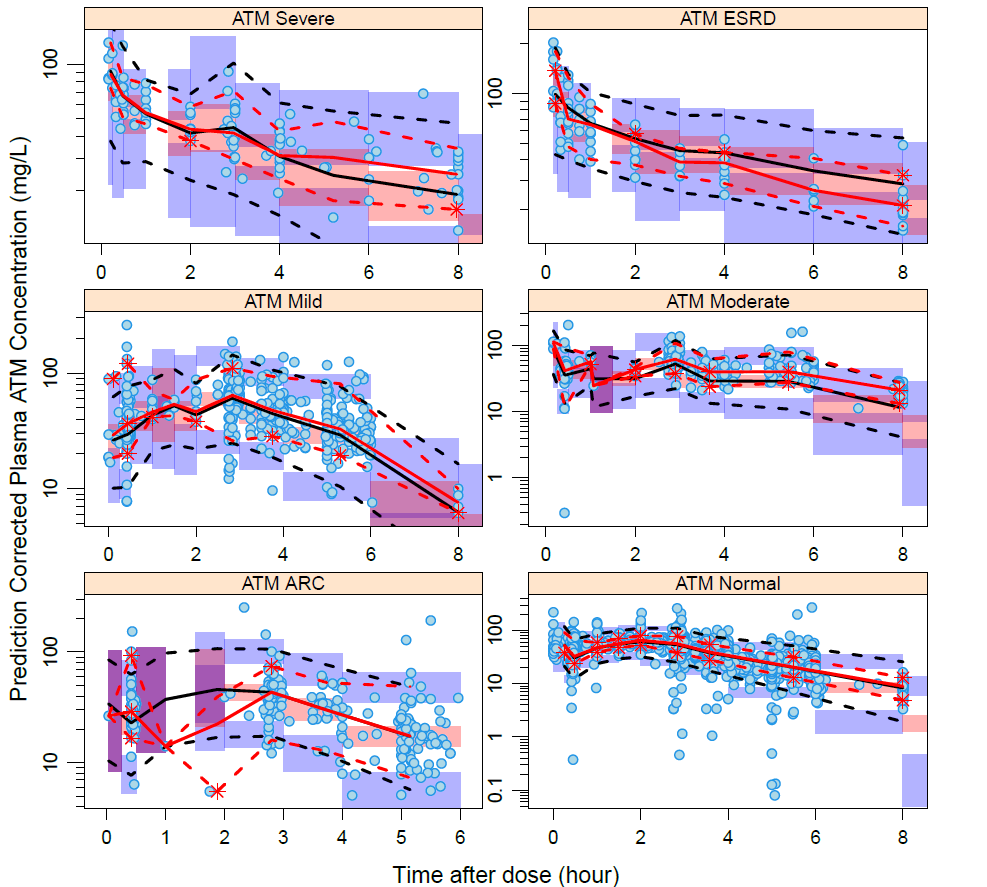


Red and black lines are the 5th, 50th (solid) and 95th percentile of observed and simulated data, respectively. Blue dots are observed data. Shadow areas are 95% confidence interval (CI) for the 5th, 50th (red) and 95th percentile prediction intervals based on simulated data. x-axis is truncated to 8 hours.

ATM = aztreonam; ARC = augmented renal clearance; ESRD = end-stage renal disease (defined as creatinine clearance ≤15 mL/min).

Figure S6. Prediction corrected visual predictive check stratified by renal function category versus time after dose for the final model for avibactam


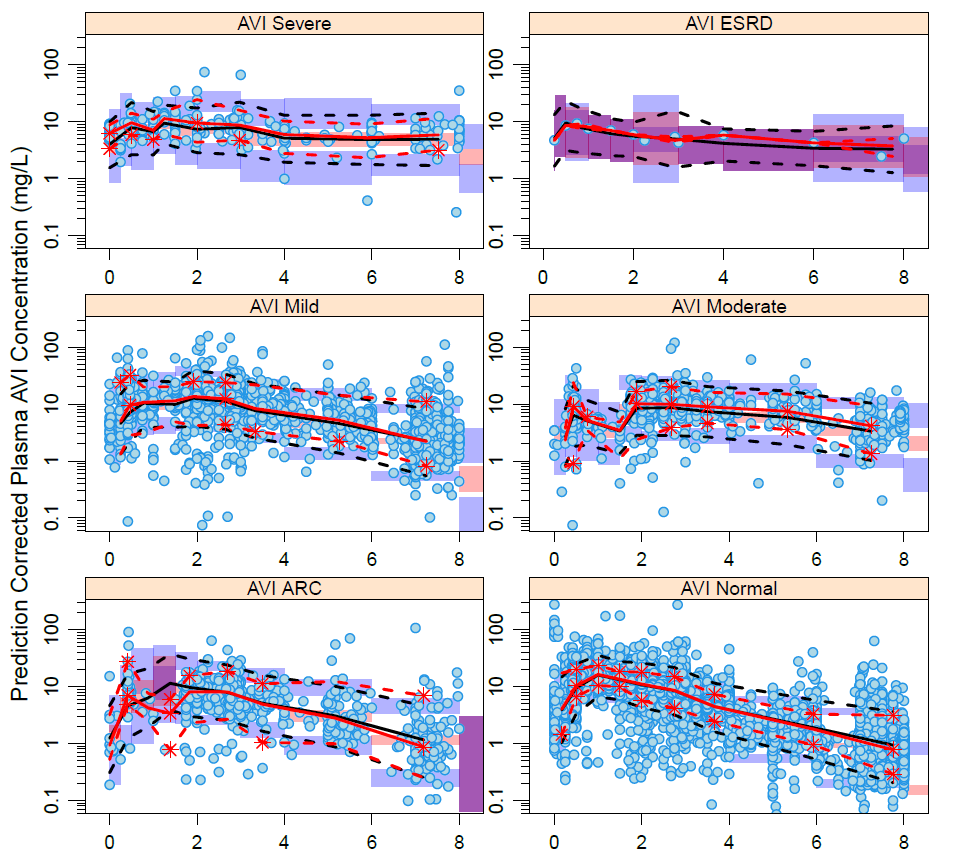


Red and black lines are the 5th, 50th (solid) and 95th percentile of observed and simulated data, respectively. Blue dots are observed data. Shadow areas are 95% confidence interval (CI) for the 5th, 50th (red) and 95th percentile prediction intervals based on simulated data. x-axis is truncated to 8 hours.

ARC = augmented renal clearance; AVI = avibactam; ESRD = end-stage renal disease (defined as creatinine clearance ≤15 mL/min).
